# ROS-scavenging lipoic acid-modified chitosan hydrogels with rapid photocrosslinked ability accelerates peripheral nerve regeneration

**DOI:** 10.1093/rb/rbag068

**Published:** 2026-04-03

**Authors:** Xianglong Chen, Ren Gao, Xinyue Liang, Kun Liu, Zhong Wei, Xiaopei Wu, Takashi Goto, Chengjie Xiong, Feng Xu, Honglian Dai

**Affiliations:** State Key Laboratory of Advanced Technology for Materials Synthesis and Processing, and Hubei Key Discipline Laboratory of Orthopedic Tissue Injury and Repair, Wuhan University of Technology, Wuhan 430070, China; Wuhan University of Technology Advanced Engineering Technology Research Institute of Zhongshan City, Zhongshan 528400, China; The First School of Clinical Medicine, Southern Medical University, Guangzhou 510515, China; Department of Orthopaedics, General Hospital of Central Theater Command, Wuhan 430070, China; State Key Laboratory of Advanced Technology for Materials Synthesis and Processing, and Hubei Key Discipline Laboratory of Orthopedic Tissue Injury and Repair, Wuhan University of Technology, Wuhan 430070, China; State Key Laboratory of Advanced Technology for Materials Synthesis and Processing, and Hubei Key Discipline Laboratory of Orthopedic Tissue Injury and Repair, Wuhan University of Technology, Wuhan 430070, China; Department of Orthopaedics, General Hospital of Central Theater Command, Wuhan 430070, China; State Key Laboratory of Advanced Technology for Materials Synthesis and Processing, and Hubei Key Discipline Laboratory of Orthopedic Tissue Injury and Repair, Wuhan University of Technology, Wuhan 430070, China; State Key Laboratory of Advanced Technology for Materials Synthesis and Processing, and Hubei Key Discipline Laboratory of Orthopedic Tissue Injury and Repair, Wuhan University of Technology, Wuhan 430070, China; The First School of Clinical Medicine, Southern Medical University, Guangzhou 510515, China; Department of Orthopaedics, General Hospital of Central Theater Command, Wuhan 430070, China; The First School of Clinical Medicine, Southern Medical University, Guangzhou 510515, China; Department of Orthopaedics, General Hospital of Central Theater Command, Wuhan 430070, China; State Key Laboratory of Advanced Technology for Materials Synthesis and Processing, and Hubei Key Discipline Laboratory of Orthopedic Tissue Injury and Repair, Wuhan University of Technology, Wuhan 430070, China; Wuhan University of Technology Advanced Engineering Technology Research Institute of Zhongshan City, Zhongshan 528400, China

**Keywords:** peripheral nerve regeneration, injectable hydrogel, α-lipoic acid, anti-inflammatory

## Abstract

Peripheral nerve injury (PNI) triggers excessive oxidative stress and inflammation that impede nerve regeneration, leading to target organ atrophy and incomplete functional recovery. Conventional drug-loaded nerve conduits release anti-inflammatory drugs sustainably via filled hydrogels, requiring a precisely engineered hydrogel system to match drug release kinetics without impeding regenerating nerve tissue growth. Meanwhile, constructing complex conduit systems may introduce potential cytotoxic factors and induce aseptic inflammation via acidic degradation products. Herein, we fabricated a ROS-scavenging CS-LA/P(MMD-CL) composite nerve conduit with rapid photo-crosslinking (365 nm UV irradiation, photoinitiator-free) via integrating lipoic acid-modified chitosan (CS-LA) hydrogel into an oriented P(MMD-CL) conduit. Disulfide bonds in CS-LA scavenged ROS to alleviate oxidative stress, modulate inflammatory factor expression and resolve excessive inflammation, while the oriented P(MMD-CL) provided physical support and directional guidance for axon growth. *In vivo* studies on SD rats revealed that CS-LA/P(MMD-CL) promoted nerve regeneration through enhancing cell proliferation and angiogenesis, improving the maturity of regenerative peripheral nerve, mitigating gastrocnemius atrophy and promoting nerve function recovery (SFI: −77.51 ± 1.51), and exhibited great biosafety—no organ damage from degradation byproducts. This simple, multifunctional composite conduit effectively modulates the PNI microenvironment and promotes peripheral nerve regeneration, offering a promising strategy for PNI repair.

## Graphical abstract

**Graphical Abstract:**
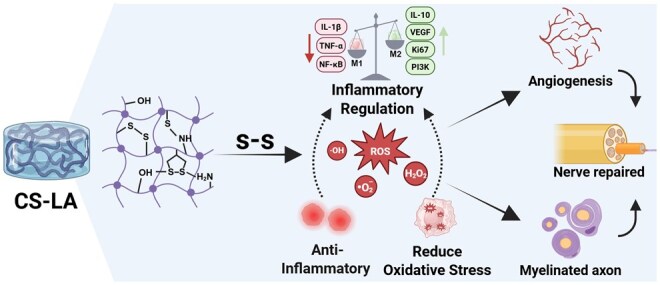

## Introduction

PNI refers to the damage caused by accidents or iatrogenic injuries that lead to nerve dysfunction, even necrosis, which results in a series of problems, including blocked or delayed nerve signals and loss of motor function and feeling. While peripheral nerves have a certain degree of self-regeneration ability, the repair process is time-consuming, resulting in considerable sequelae such as muscle atrophy and incomplete functional recovery [1–3]. It can be attributed to the major challenges in the early stage, for example, excessive inflammatory response and oxidative stress, vascular damage, mitochondrial damage, etc. [4]. Reactive oxygen species (ROS), an important regulatory signal molecule in life processes, participate in numerous signal transduction cascades, and directly affect the processes of cell proliferation, differentiation and apoptosis [5]. In the early stage of PNI, as a part of the body’s self-defense mechanism, the mitochondria of damaged tissue cells initiate the apoptosis program, releasing a large amount of ROS, causing an oxidative stress microenvironment and inflammatory response, which in turn affects the physiological behavior of Schwann cells and the immune system (mainly macrophages) [6, 7]. However, the ROS released during this process is often excessive, which greatly hinders the transition of the regeneration process from anti-inflammation to regenerating axon growth and extension [8]. In addition to nerve anastomosis and autologous nerve transplantation, nerve guidance conduit (NGC) is a promising alternative method with great compatibility and strong designability [9, 10]. NGCs can be designed to regulate the regenerative microenvironment while providing physical support and directional guidance for regenerative axons [11]. However, traditional NGC itself doesn’t have the function of eliminating excessive ROS to alleviate oxidative stress—even the novel P(MMD-CL) NGC we developed previously [12]. To expand its functionality, strategies such as multi-block copolymerization or constructing a composite system are often necessary [13, 14]. Meanwhile, NGCs prepared with synthetic polymers, such as poly-ε-caprolactone (PCL), poly(L-lactic acid) (PLLA), poly(glycolic acid) (PGA), etc., will slowly release acidic molecules during the degradation process, which is prone to causing aseptic inflammation at the implantation site [15]. Therefore, there is an urgent need to develop an NGC composite system capable of eliminating excessive ROS, with degradation products that do not induce secondary inflammation. Under such circumstances, filling NGC with hydrogel that possesses ROS-scavenging capability emerges as a more effective and viable strategy—one that has been widely validated and applied [16–18].

As a natural polymer formed by deacetylation of chitin, chitosan exhibits outstanding biological characteristics, including superior biocompatibility, biodegradability, low toxicity and antibacterial properties. Accordingly, it has gained widespread applications in biomedical areas such as tissue engineering, drug delivery and wound healing materials [19, 20]. Studies have shown that the chitosan degradation product, amino sugar, can be completely absorbed by the body without negative effects on the regeneration microenvironment [21]. Besides, chitosan-based hydrogels have similar composition and mechanical properties to the extracellular matrix, which can promote cell adhesion, growth and migration, and provide extra structural support to NGCs, making them potential candidates for NGC fillers [22]. However, chitosan exhibits poor water solubility in neutral and alkaline solutions. A common strategy is to use acrylic or methacrylic modified chitosan with better water solubility for further application, but an additional photoinitiator is needed, which may introduce potential cytotoxicity [23]. Therefore, there is a need for a chitosan hydrogel that can be easily cross-linked and effectively remove excessive ROS, promoting peripheral nerve regeneration.

α-Lipoic acid (LA) is a powerful natural antioxidant produced in mitochondria. Its closed five-membered ring structure has a high electron density, giving it significant electrophilic properties, enabling it to react with free radicals like ROS and reactive nitrogen species [24–27]. However, LA is a hydrophobic molecule [28]. Thus, we grafted lipoic acid molecules onto chitosan via an amidation reaction. Under 365 nm UV irradiation, the lipoic acid-modified chitosan forms a new crosslinking network through the cleavage and reconstruction of disulfide bonds (S-S), yielding a chitosan hydrogel with ROS-scavenging capability.

**Figure 1:**
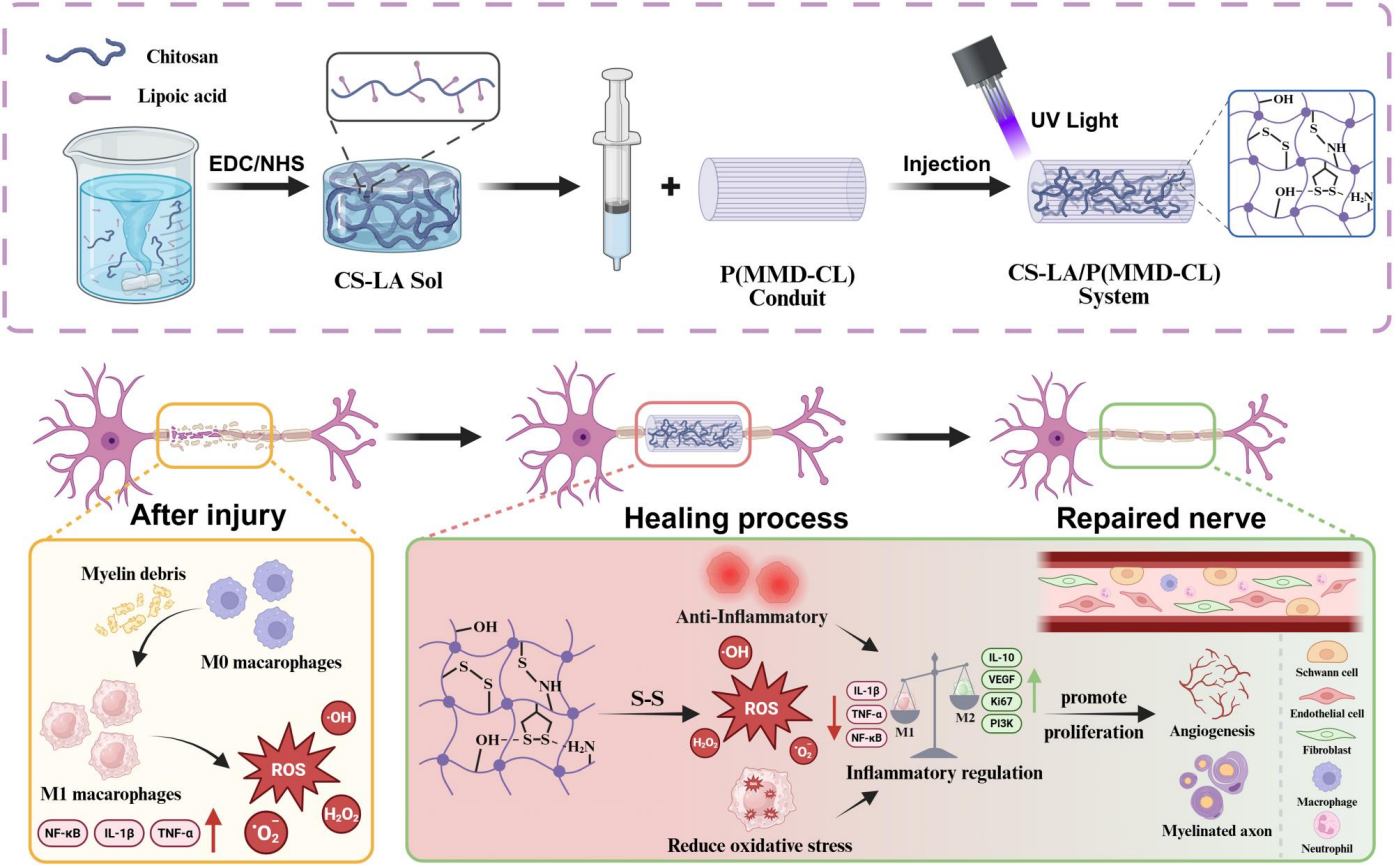
Schematic illustration of the formation for CS-LA/P(MMD-CL) conduit. After PNI, apoptosis and the immune system trigger excessive ROS release, creating a microenvironment of oxidative stress and excessive inflammation. The S-S of CS-LA hydrogel reacts with and scavenges ROS, thereby downregulating pro-inflammatory factors expression and upregulates restorative factors (e.g. IL-10, VEGF) secretion, which initiates the PI3K/AKT signaling cascade to accelerate cell proliferation and angiogenesis, provide nutrient support, and ultimately promote axonal regeneration and motor function recovery.

Here, we hypothesized that the CS-LA hydrogel, integrated into an oriented P(MMD-CL) conduit, would effectively promote peripheral nerve regeneration via integrated management: concurrently enabling photoinitiator-free crosslinking and targeting the PNI-associated oxidative and inflammatory microenvironment. To test this, we developed CS-LA/P(MMD-CL) composite conduit (Figure 1). After implantation, the S-S reacted with excessive ROS, alleviating oxidative stress and thereby modulating the expression of inflammatory factors to exert an anti-inflammatory effect and further promote angiogenesis. Concurrently, the cleavage of S-S post-reaction accelerated hydrogel degradation, allowing regenerating nerve axons to grow faster under the guidance of oriented P(MMD-CL) fibers. Notably, oxidative stress and excessive inflammation accompany most traumatic injuries in the early post-injury phase—suggesting that the CS-LA hydrogel may provide a novel strategy for a broader range of traumatic injuries.

## Materials and methods

### Materials

Chitosan (viscosity-average molecular weight: 200 kDa; ≥95% deacetylation) was purchased from Shanghai Macklin Biochemical Co., Ltd. Morpholine-2,5-dione (MMD) was synthesized using the previous method [12]. N-hydroxysuccinimide (NHS), 1, 1, 1, 3, 3, 3-hexafluoro-2-propanol, (1-(3-dimethylaminopropyl)-3-ethylcarbodiimide hydrochloride) (EDC), L-Alanine (Ala) was supplied by Shanghai Aladdin Biochemical Technology Co., Ltd. Sn(Oct)_2_, ε-caprolactone and methacrylic anhydride were purchased from Sigma-Aldrich. (R)-α-lipoic acid was obtained from Tokyo Chemical Industry (TCI, Shanghai). Triethylamine, CH_2_Cl_2_, CHCl_3_, N, N-dimethylformamide (DMF), methanol, acetic acid (CH_3_COOH), chloroacetyl chloride, NaOH, HCl, were obtained from Sinopharm Chemical Reagent (Beijing Co. Ltd, China). All other reagents and solvents were of analytical grade and used as received without additional purification.

### Synthesis of chitosan modified with lipoic acid

Chitosan (0.6 g) was dispersed in 30 mL 1.5% (v/v) aqueous acetic acid solution and stirred for 1 h until completely dissolved, yielding 2% CS solution (w/v). LA was dissolved in 40 mL of ethanol and added dropwise into the CS solution at varied molar ratios (1/20, 1/10, 1/5), denoted as CS-LA 1/20, CS-LA 1/10 and CS-LA 1/5, respectively. The mixture was stirred at room temperature until homogeneous. Then, 0.8 g EDC and 0.2 g NHS were introduced to catalyze the reaction for 4 h. The final product was purified by dialysis for 5 days and lyophilized for further use.

### Synthesis of P(MMD-CL)

Briefly, 10 g ε-caprolactone, 1 g lab-synthesized MMD and 5.5 mL 1% (w/v) Sn(Oct)_2_ solution in anhydrous CHCl_3_ were added into a polymerization tube. Then the device was frozen in liquid nitrogen and evacuated to remove air. After 15 min, it was filled with nitrogen and transferred to a 35°C water bath until fully thawed. This cycle of freezing-evacuation-nitrogen filling-thawing was repeated three times to ensure an oxygen-free system. The polymerization device was then vacuum-sealed and reacted at 130°C in an oil bath for 24 h. The crude product was dissolved in DCM, precipitated in excess methanol and this dissolution-precipitation process was repeated three times for further use.

### Characterization of CS-LA

Chemical structure of the hydrogels was characterized by Fourier transform infrared spectroscopy (FT-IR, Waltham, MA, USA) and UV-Vis-NIR (Lambda 750 S, USA). Rheological analysis was used to determine the gelation time of the CS-LA solution. Each product was observed by scanning electron microscopy (SEM, JSM-IT300, Japan). The freeze-dried CS-LA hydrogels were analyzed using UNICUBE (CHNSO) elemental analyzer (Elementar, Germany) to determine the contents of C, H, N and S elements, and further calculated the grafting ratio (GR) of lipoic acid on chitosan. *W_s_* is the percentage of *S* element in total mass, *W_c_* is the percentage of *C* element in total mass. The grafting amount of lipoic acid was calculated using the following formula:


(1) $$\mathrm{GR}=\frac{7200\;*\;W_{s}}{64\;*\;W_{c}-96*W_{s}}$$

A gravimetric method reported previously was used to measure the swelling ratio of the CS-LA hydrogel [29], and the degradation properties were carried out with 0.01 M PBS. Specifically, CS-LA hydrogel cylinders (5 mm thickness, 10 mm diameter) were fabricated using custom-made molds. After accurately weighing the initial mass (m_0_), each cylinder was individually placed into a sealed centrifuge tube containing 10 mL of 0.01 M PBS. At predetermined intervals (1, 3, 5, 7, 14, 21 and 28 days), samples were retrieved, removing surface moisture and immediately weighed to obtain the residual mass (*m_t_*). Three parallel samples were tested per group for each interval. The mass loss rate was calculated by the following formula:


(2) $$\mathrm{Mass}\;\mathrm{loss}\;\mathrm{rate}\;(\%)=\frac{m_{0}-m_{t}}{m_{t}}\times 100\%$$

### Characterization of P(MMD-CL)

^1^H NMR spectra were recorded on an Avance III HD 500 MHz NMR spectrometer (Bruker, Germany). Chemical structure was characterized by FT-IR (Waltham, MA, USA). A contact angle meter (FACE CA-XP150, Powereach, China) was used to measure the hydrophilicity of both P(MMD-CL) and PCL fiber membranes, and degradation was conducted in 0.01 M PBS. In brief, 0.1 g of electrospun conduit was incubated with 10 mL of PBS in a centrifuge tube at 37°C with shaking at 100 rpm. Weight and pH were monitored for 8 weeks, with samples collected and tested weekly, and fresh PBS was re-added accordingly.

### Mechanical analysis of CS-LA and P(MMD-CL) and rheological analysis of CS-LA

Cylindrical CS-LA hydrogels were prepared, and a universal testing machine (Instron 5967, China) was used to measure the mechanical properties of hydrogels and fiber membranes. Hydrogels were compressed at 1 mm min^−1^, and fiber membranes were tested at 100 mm min^−1^ to acquire a stress-strain curve (*n* = 3). Rheometer (Malvern Kinexus Pro KNX2100) was used to perform rheological behavior of CS-LA hydrogels.

### The scavenging of DPPH and ·OH

1,1-Diphenyl-2-picrylhydrazyl (DPPH) was dissolved in anhydrous methanol to prepare a 400 μmol/L DPPH solution. Subsequently, hydrogel cylinder samples were divided into experimental, control and blank groups. Each sample was mixed with 3.5 mL DPPH solution and incubated at 37°C with shaking at 100 rpm in darkness for 12 h. During incubation, 100 μL supernatant was collected hourly into a 96-well plate, and absorbance at 517 nm was measured using a microplate reader (Multiskan GO-1510, ThermoFisher, USA), where the absorbance of the experimental group was denoted as *A_s_*, the control group as *A_c_* and the blank group as *A_b_*. DPPH clear rate was calculated as follows:


(3) $$\mathrm{DPPH}\;\mathrm{clear}\;\mathrm{rate}\;(\%)=(1-\frac{A_{s}-A_{c}}{A_{b}})\times 100\%$$

80 mg FeSO_4_·7H_2_O and 40 mL 30% H_2_O_2_ were separately dissolved in deionized water to prepare 120 mL solutions. Subsequently, the two solutions were mixed and reacted for 10 min to generate hydroxyl radical (·OH). The subsequent experimental procedures were consistent with those of the aforementioned DPPH scavenging assay, except that 100 μL of the supernatant was collected every 0.5 h into a 96-well plate. Then, 25 μL of 0.01 M salicylic acid ethanol solution was added into each well, and oscillated gently for 10 min to ensure sufficient reaction. After that, the absorbance at 510 nm was measured using the same microplate reader, where the absorbance of the experimental group was denoted as *A_e_*, the control group as *A_t_* and the blank group as *A_k_*. ·OH clear rate was calculated as follows:


(4) $$\cdot \mathrm{OH}\;\mathrm{clear}\;\mathrm{rate}\;(\%)=(1-\frac{A_{e}-A_{t}}{A_{k}})\times 100\%$$

### *In vitro* studies

#### Cytotoxicity and cell proliferation test

Cytotoxicity and cell proliferation were evaluated by CCK-8 assay (NCM Biotech, C6005, Suzhou, China). RSCs and HUVECs were incubated with various complete hydrogel extract media for 1, 3 and 5 days, with a blank control set. After incubation, the medium was replaced with 10% CCK-8 reagent solution and incubated for 30 min. Microplate reader (Multiskan GO-1510, ThermoFisher, USA) was used to measure absorbance at 450 nm. Cell viability was assessed via LIVE/DEAD staining. RSCs cultured with different extracts for 1, 3 and 5 days were stained with Calcein-AM (Yeasen, China) and propidium iodide (Yeasen, China) in darkness for 30 min. Cells were observed under a fluorescence microscope (Olympus IX71, Japan).

#### Study on promoting angiogenesis

##### Migration assay

Suspended HUVECs were seeded into 6-well plates at 1 × 10^6^ cells per well and cultured at 37°C under 5% CO_2_ atmosphere until reaching 80–90% confluence. A linear scratch was made in the cell monolayer using a 10 μL pipette tip, and loose cells were gently rinsed away with PBS. Different complete hydrogel extract media were then added, and cells were cultured for 24 h. Migrated HUVECs into the scratched area were photographed at 0, 12 and 24 h. The wound healing ratio was analyzed using ImageJ software (v1.8.0).

##### Transwell

HUVECs in the logarithmic growth phase were cultured in serum-free medium for 12 h, then seeded into the upper chamber at 2 × 10^5^ cells per well with 200 μL 1% FBS DMEM. About 600 μL of different complete hydrogel extract media was added to the lower Transwell chamber (3422, Corning). Cells were incubated at 37°C under 5% CO_2_ for 24 h. Afterwards, non-migrated cells in the upper chamber were removed with a cotton swab, and the membrane was washed twice with cold PBS. After fixation in 4% paraformaldehyde for 30 min and staining with 0.1% crystal violet (G1063, Solarbio) for 20 min, the membrane was washed three times with cold PBS. Migrated cells on the lower surface were photographed using an inverted microscope (IX73, Olympus, Japan).

##### Tube formation assay

HUVECs were co-cultured with different complete hydrogel extract media in culture flasks for 7 days, after which cell suspensions were prepared separately. Matrigel (356234, Corning) was added to pre-cooled 24-well plates (100 μL per well) and incubated at 37°C for 30 min. HUVECs were seeded at 2 × 10^4^ cells per well. All consumables used in this process, such as pipette tips, were pre-cooled in a −20°C refrigerator. After 6 h incubation, tube formation was observed under an inverted microscope (IX73, Olympus, Japan).

#### Antioxidant and anti-inflammatory properties

##### Antioxidant immunofluorescence

RSCs were seeded in 6-well plates and cultured for 24 h at 37°C under 5% CO_2_. The medium was then replaced with complete medium supplemented with different hydrogel extracts, followed by a further incubation for 24 h. After that, the cells were washed twice with PBS, and 400 μM H_2_O_2_-containing DMEM was added to induce intracellular ROS generation. ROS levels were detected using a DCFH-DA assay kit (CA 1410, Solarbio, China). Following H_2_O_2_ treatment, RSCs were incubated with 10 μM DCFH-DA at 37°C in darkness for 20 min, then washed twice with PBS. Fluorescence signals (excitation 488 nm) were visualized and imaged with a microscope (IX73, Olympus, Japan); images were captured for qualitative and semi-quantitative analysis of ROS production.

##### Detection of MMP

MMPs were assessed through the JC-1 MMP detection kit (M8650, Solarbio, China). After the aforementioned treatments, RSCs were stained with the JC-1 working solution at 37°C in darkness for 20 min. The supernatant was then carefully removed, and cells were washed twice with 1× JC-1 staining buffer to remove excess dye. Fresh DMEM was added to each well, and the stained cells were imaged immediately by an inverted fluorescence microscope (Model IX73, Olympus, Tokyo, Japan). Red fluorescence indicated high MMP, while green fluorescence indicated low MMP.

##### Anti-inflammatory immunofluorescence

Immunofluorescence was used to evaluate CS-LA hydrogel effects on cell lines under inflammatory conditions. RAW 264.7 cells were seeded at 1 × 10^6^ cells per plate and cultured in an incubator for 24 h. After two PBS washed, cells were incubated in 500 ng mL^−1^ LPS-containing DMEM for 12 h. Following another two PBS washes, the medium was replaced with complete medium supplemented with different hydrogel extracts, and cells were co-cultured with hydrogels for 2 days. Cells were then fixed with paraformaldehyde and stained with NF-κB and IL-1β antibodies to observe the inflammatory response.

### *In vivo* studies

#### Animal experiment

A 12‑week rat sciatic nerve defect model was employed to evaluate the nerve regeneration ability of CS-LA/P(MMD-CL) conduits. A total of 50 SPF male SD rats (≈200 g) were obtained from Wuhan Wan Qian Jia Xing Biotechnology Co., Ltd. And acclimated for 1 week (4 rats per cage) with free access to water and food. Rats were randomly divided into five groups (*n* = 10 each): Autograft, CS-LA 1/20, CS-LA 1/10, CS-LA 1/5 and CS-MA. All surgical procedures involving SD rats were approved by the Ethics Committee of Wuhan Myhalic Biotechnological Co., Ltd. (HLK-20230630-001) and performed following institutional animal care guidelines. The surgical procedure is shown in Supplementary Figure S10.

#### Histological analysis

At 12 weeks post-surgery, bilateral gastrocnemius muscles were harvested and weighed. The ratio of the surgical-side to normal-side gastrocnemius weight was calculated to assess muscle atrophy induced by nerve injury. Implanted conduits were then dissected, fixed in paraformaldehyde for 48 h, embedded in paraffin, and sectioned both longitudinally and transversely. Following slice preparation, samples were stained with hematoxylin and eosin (H&E), Luxol fast blue (LFB) and toluidine blue (TB) following the manufacturer’s protocols. For TEM analysis, samples were fixed with an electron microscope fixative (G1102, Servicebio). After gradient ethanol dehydration, samples were embedded in epoxy resin, cut into 80-nm ultrathin sections, and stained with lead citrate and uranyl acetate. Axonal regeneration and myelin sheath formation were observed using a JEM-1400 Plus TEM (JEOL, Japan). Meanwhile, the hearts, spleens, livers and kidneys of rats were harvested, fixed in paraformaldehyde, paraffin-embedded, sectioned and H&E-stained to evaluate the effects of different materials.

#### Immunofluorescence staining

Paraffin sections were dewaxed and rinsed sequentially. Then, the sections were placed in a pressure cooker together with EDTA antigen retrieval solution (pH = 9.0) for antigen retrieval. After retrieval, the sections were rinsed with Tris-buffered saline (TBS) buffer. Next, the sections were immersed in 3% H_2_O_2_ solution for endogenous peroxidase blocking at room temperature. Following blocking, the sections were removed and rinsed with distilled water, then transferred to TBS buffer. A 10% goat serum solution was added dropwise onto the sections, which were then incubated at 37°C for 30 min. After incubation, the goat serum was discarded by gentle tapping. The sections were incubated with the primary antibody working solution at 4°C overnight. On the following day, the sections were removed from 4°C and allowed to rewarm to room temperature. After rewarming, the sections were rinsed with TBS buffer, and the secondary antibody working solution was added dropwise. The sections were then incubated at 37°C for 45 min, followed by another rinse with TBS buffer. Subsequently, the processed sections were stained with the following antibodies: CD31 (1:8000, Rabbit monoclonal antibody, ab182981, Abcam), VEGF (1:1000, Rabbit monoclonal antibody, CY5096, Abways), NF-κB (1:1000, Rabbit monoclonal antibody, ab32536, Abcam), S100β (1:500, Rabbit monoclonal antibody, S0B2117, Starter), NF200 (1:200, Guinea pig polyclonal antibody, OB-PGP081-02, Oasis biofarm), Ki67 (1:200, Rabbit monoclonal antibody, 9129, CST), IL-1β (1:1000, Rabbit polyclonal antibody, ab283818, Abcam).

#### Behavioral assessment

Sciatic nerve functional recovery in SD rats was assessed via walking track analysis at the 12th week post-surgery. Briefly, the hindlimbs of rats (normal side [non] and injured side [exp]) were dyed, and rats crawled through a customized tunnel. After collecting footprints, podogram length (PL), toe width (TW) and mid-toe width (MTW) were measured, and the sciatic functional index (SFI) was quantified using following formula:


(5) $$\mathrm{SFI}=38.3\times \frac{{\mathrm{PL}}^{\exp}-{\mathrm{PL}}^{\mathrm{non}}}{{\mathrm{PL}}^{\mathrm{non}}}+109.5\times \frac{{\mathrm{TW}}^{\exp}-{\mathrm{TW}}^{\mathrm{non}}}{{\mathrm{TW}}^{\mathrm{non}}}+13.3\times \frac{{\mathrm{MWT}}^{\exp}-{\mathrm{MTW}}^{\mathrm{non}}}{{\mathrm{MTW}}^{\mathrm{non}}}-8.8$$

### Statistical analysis

Data were processed and analyzed using Origin 2022. Normality and variance homogeneity were verified before statistical analysis. One-way ANOVA was used for intergroup comparisons, and Tukey’s HSD *post hoc* test was employed for pairwise analysis when significance (*P* < 0.05) was detected. All tests were two-sided, with *P* < 0.05 regarded as statistically significant (*P < 0.05, **P < 0.01, ***P < 0.001, ****P < 0.0001).

## Results and discussion

### Synthesis and characterization of CS-LA/P(MMD-CL)

**Figure 2:**
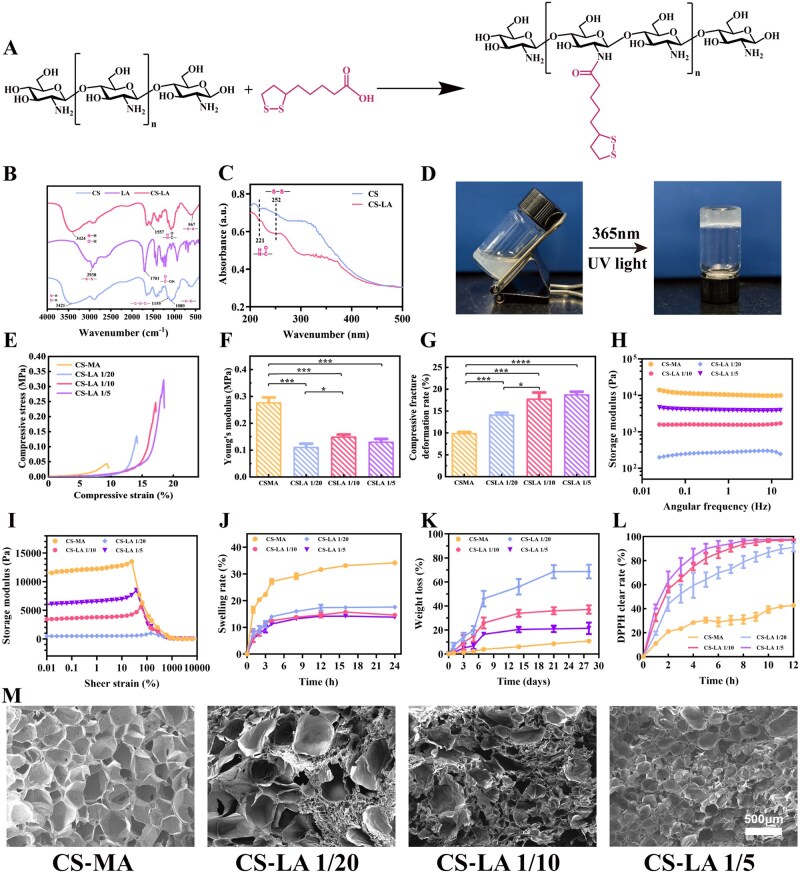
Synthesis mechanism and characterization of CS-LA hydrogel. (**A**) Schematic illustration of CS-LA synthesis mechanism. (**B**) FI-IR spectra of CS, LA and CS-LA. (**C**) UV spectra of CS and CS-LA. (**D**) Performance of CS-LA sol-gel transition with 365 nm UV light crosslinking. (**E**) The compressibility of CS-LA and CS-MA hydrogel. (**F, G**) Young’s modulus and ultimate tensile strain of CS-LA and CS-MA hydrogel (*n* = 3). (**H**) Frequency sweep measurements of hydrogels. (**I**) Storage modulus (G′) curves from rotational strain sweeps of hydrogels. (**J**) Swelling rate of CS-LA and CS-MA hydrogels (*n* = 3). (**K**) The weight loss rate of CS-LA and CS-MA hydrogels within 28 days (*n* = 3). (**L**) DPPH clear rate of CS-LA and CS-MA hydrogels (*n* = 3). (**M**) The microstructure of CS-LA hydrogels. Scale bar: 500 μm. *P < 0.05, **P < 0.01, ***P < 0.001, ****P < 0.0001.

Chitosan was modified with lipoic acid via an amide reaction under the catalysis of EDC/NHS in this research (Figure 2A). Methacrylic chitosan (CS-MA) was synthesized using the previous method as a control group [30] (Supplementary Figure S1A–C). The successful synthesis of CS-LA was jointly confirmed by FT-IR (Figure 2B) and ultraviolet (UV) absorption spectroscopy (Figure 2C). In the FT-IR spectrum of CS-LA, a distinct absorption peak at 1557 cm^−1^ was observed, which is attributed to the in-plane bending vibration of amide bonds, confirming the occurrence of an amide condensation reaction between CS and lipoic acid. Additionally, CS-LA exhibited a unique peak at 567 cm^−1^, which can be assigned to the stretching vibration of S-S [31]. Complementary ultraviolet-visible spectroscopic analysis revealed two specific absorption peaks for CS-LA: a peak at 252 nm corresponding to S-S and another peak at 221 nm arising from the amide bond, whereas no such characteristic peak was detected in pristine CS [32]. As the molar ratio of LA to CS increased from 1/20 to 1/5, the grafting ratio of lipoic acid onto CS-LA increased from 1.37% to 9.85% (Supplementary Table S3), and the color of the CS-LA solution gradually deepened to an opalescent white (Supplementary Figure S2A). Photographs of various CS-LA hydrogels are displayed in Supplementary Figure S2B, while the WUT writing of CS-LA hydrogels is shown in Supplementary Figure S2C, exemplifying distinct appearance characteristics and plasticity. Notably, the CS-LA solution was able to be converted into a hydrogel under mild conditions via irradiation with 365 nm ultraviolet light (Figure 2D), which was attributed to the cleavage and reconstruction of S-S in lipoic acid, enabling the construction of a crosslinked hydrogel network (Figure 1) [33]. Rheological characterization revealed that an increase in the grafting ratio of LA onto CS shortened the gelation time and enhanced the storage modulus (G′) (Supplementary Figure S3A and B), with G′ consistently greater than the loss modulus (G″). The shortened gelation time is particularly favorable for the biomedical application of the CS-LA hydrogel, as it minimizes potential cell damage caused by prolonged UV exposure.

Compressive stress–strain curves (Figure 2E) demonstrated that, compared with CS-MA hydrogels, CS-MA exhibited a higher Young’s modulus than CS-LA (Figure 2F). The CS-MA hydrogel fractured at a compression of 9.40%, whereas the CS-LA hydrogel sustained compression up to 14.13% and could withstand a maximum compressive deformation of 18.44% (Figure 2G), indicating that the CS-LA hydrogel is a soft yet tough hydrogel material. The rheological properties of the CS-LA hydrogel were further characterized. Frequency sweep measurements of the storage modulus (G′) (0.01–100 rad s^−1^, 1% strain) (Figure 2H) revealed that the G′ value of the CS-LA hydrogel was independent of angular frequency, confirming the formation of a stable and dense crosslinked network. Strain sweep results (0.01–10 000%, 10 rad s^−1^) (Figure 2I) showed that the shear strain tolerance of the CS-LA hydrogel increased with the molar ratio of LA to CS. This verified that the introduction of more LA facilitated the formation of a denser crosslinked network. The swelling and degradation behaviors of the CS-LA hydrogel were investigated by immersing the hydrogels in PBS at 37°C. Both hydrogels reached swelling equilibrium at a comparable rate, and CS-MA hydrogels exhibited a significantly higher swelling ratio (34.16%). In contrast, the final swelling ratios of CS-LA 1/20, CS-LA 1/10 and CS-LA 1/5 were 17.58%, 14.57% and 13.80%, respectively (Figure 2J). After 30 days of degradation, the mass loss rates of all CS-LA hydrogels were higher than those of the CS-MA hydrogel, which was 10.82% (Figure 2K). This phenomenon could be attributed to differences in material hydrophilicity, crosslinking density and pore structure, which collectively regulate the water absorption capacity of the hydrogels. The microstructure of CS-MA and CS-LA hydrogels was observed via SEM (Figure 2M). The cross-section of CS-MA hydrogels displayed a uniformly porous network structure, whereas the CS-LA hydrogel exhibited uneven pore sizes and a relatively loose structure. With an increase in the LA molar ratio, the microstructure of the CS-LA hydrogel gradually became more regular and ordered. These properties enable CS-LA hydrogels to provide structural support for conduits and protect regenerating axons from compression. The antioxidant capacity of the CS-LA hydrogel was evaluated via DPPH radical scavenging and ·OH radical scavenging assays. When tested with a 400 μmol/L DPPH solution, both CS-LA 1/10 and CS-LA 1/5 hydrogels completely scavenged DPPH radicals within 10 h, while the CS-LA 1/20 hydrogel achieved a final scavenging rate of 90% (Figure 2L). Consistent with DPPH results, ·OH radical scavenging assay further confirmed the antioxidant activity of CS-LA hydrogels. Within 6 h, CS-LA 1/5 scavenged 91.42% of the generated ·OH radicals, CS-LA 1/10 approximately 79.43%, and CS-LA 1/20 about 61.85%, all of which were significantly higher than the 30.61% scavenging rate of CS-MA (Supplementary Figure S4).

**Figure 3:**
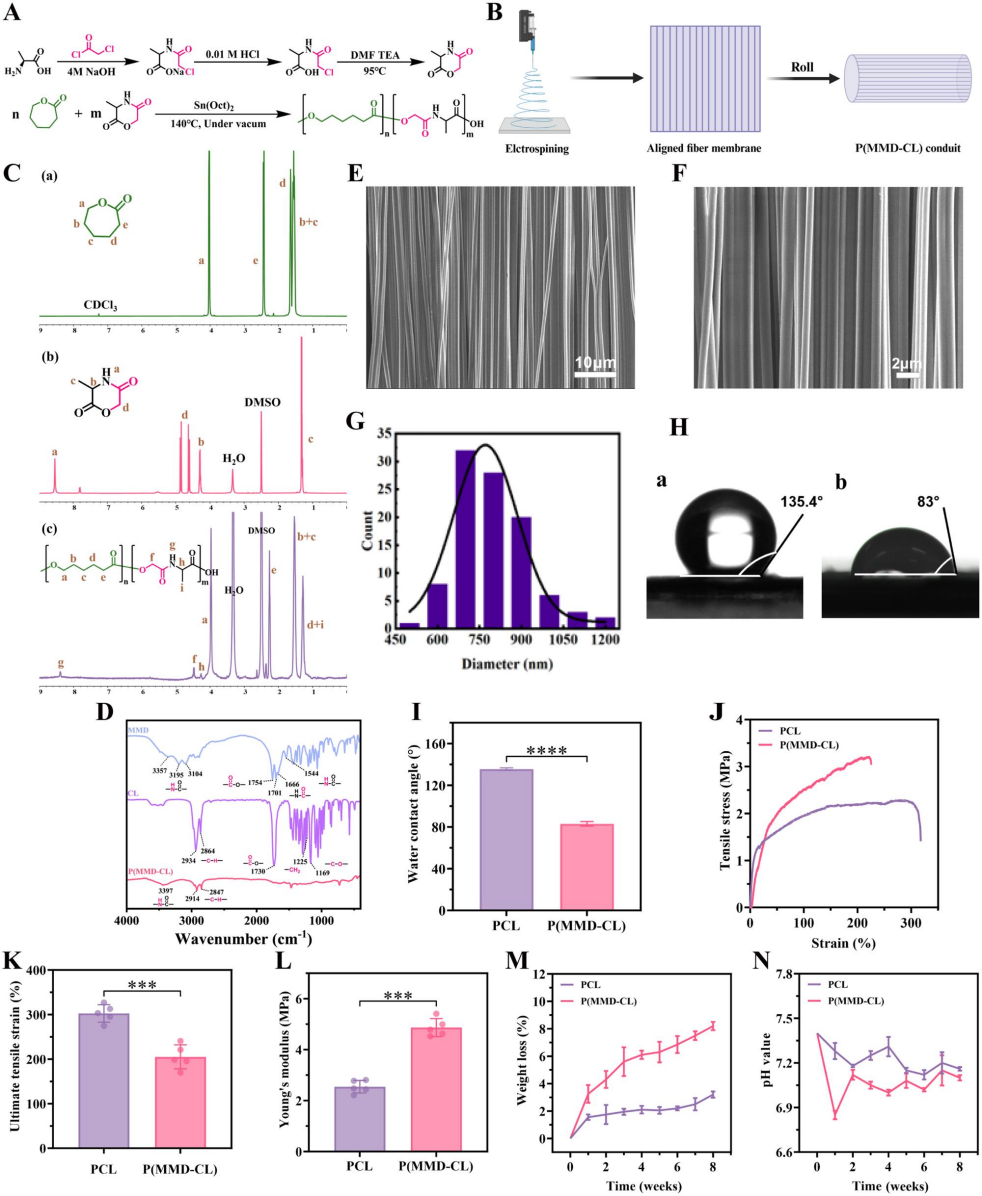
Synthesis mechanism and characterization of P(MMD-CL). (**A**) The synthesis of MMD monomer and the ring-opening polymerization reaction mechanism. (**B**) Electrospinning fiber membrane preparation images. (**C**) ^1^H NMR spectrum of CL, MMD and P(MMD-CL). (**D**) FT-IR spectra of CL, MMD, P(MMD-CL). (**E, F**) SEM images of P(MMD-CL) fiber membranes. Scale bars, 10 and 2 μm. (**G**) Diameter statistical histogram and normal distribution curve of P(MMD-CL). (**H**) Typical images of (a) PCL and (b) P(MMD-CL) water contact angle. (**I**) water contact angle (*n* = 3), (**J**) Stress–strain curves, (**K**) ultimate tensile strain (*n* = 5) and (**L**) Young’s modulus (*n* = 5) of PCL and P(MMD-CL). (**M**) Weight loss rate and (**N**) degradation pH of PCL and P(MMD-CL) within 8 weeks (*n* = 3). *P < 0.05, **P < 0.01, ***P < 0.001, ****P < 0.0001.

Following our previous report [12], P(MMD-CL) was synthesized via melt ring-opening polymerization of MMD and CL monomers, with Sn(Oct)_2_ as the catalyst (Figure 3A). Oriented fibrous membranes of PCL and P(MMD-CL) were fabricated via electrospinning for subsequent characterization (Figure 3B). Figure 3C presents the ^1^H NMR spectra of the products at each synthesis step. The synthesis process was further analyzed by FT-IR spectra (Figure 3D), which collectively confirmed the successful synthesis of P(MMD-CL). The morphology of the oriented P(MMD-CL) fibrous membrane was observed by SEM (Figure 3E and F), and the average fiber diameter was quantified via ImageJ. The P(MMD-CL) fibers exhibited an average diameter of 845.93 ± 187.12 nm, with a normal distribution of fiber diameters (Figure 3G). The water contact angle tests indicated that the hydrophilicity of P(MMD-CL) was significantly improved (Figure 3H and I). Mechanical characterization showed that P(MMD-CL) had an ultimate tensile strain of 205.04 ± 26.97% (Figure 3J and K) and a Young’s modulus of 4.87 ± 0.35 MPa (Figure 3L), which was comparable to those of PCL and sufficient to meet the need of mechanical support requirements of nerve conduits. To evaluate *in vitro* degradation behavior, the fibrous membranes were rolled into nerve conduits and immersed in PBS for an 8-week degradation test (Figure 3M). The pH of the system (Figure 3N) was monitored throughout the process. After 8 weeks, the degradation rate of PCL was only 3.2%, while that of P(MMD-CL) reached 8.2%. This higher degradation rate of P(MMD-CL) is attributed to the amide bonds in the MMD segments, which are more susceptible to cleavage and thus promote degradation. Although P(MMD-CL) degraded more rapidly, the pH of the system remained above 6.85 throughout the entire test period. In summary, compared with PCL, P(MMD-CL) exhibits enhanced hydrophilicity and degradation performance while retaining favorable mechanical properties, demonstrating that P(MMD-CL) holds promising application potential for nerve repair.

### Cytotoxicity and angiogenesis studies

Biomaterials should exhibit minimal cytotoxicity. Therefore, the *in vitro* cytotoxicity of CS-LA hydrogels was evaluated by RSCs. Standard CCK-8 assays were performed to measure cell viability on days 1, 3 and 5 after the addition of hydrogels. Neither hydrogel showed obvious cytotoxicity, with cell survival rates exceeding 95% (Supplementary Figure S5A). To further confirm the favorable cytocompatibility of the hydrogels, *in vitro* LIVE/DEAD cell staining assays were conducted. Fluorescence microscopy observations revealed no significant RSC death by day 5 of the experiment (Supplementary Figure S5B).

**Figure 4:**
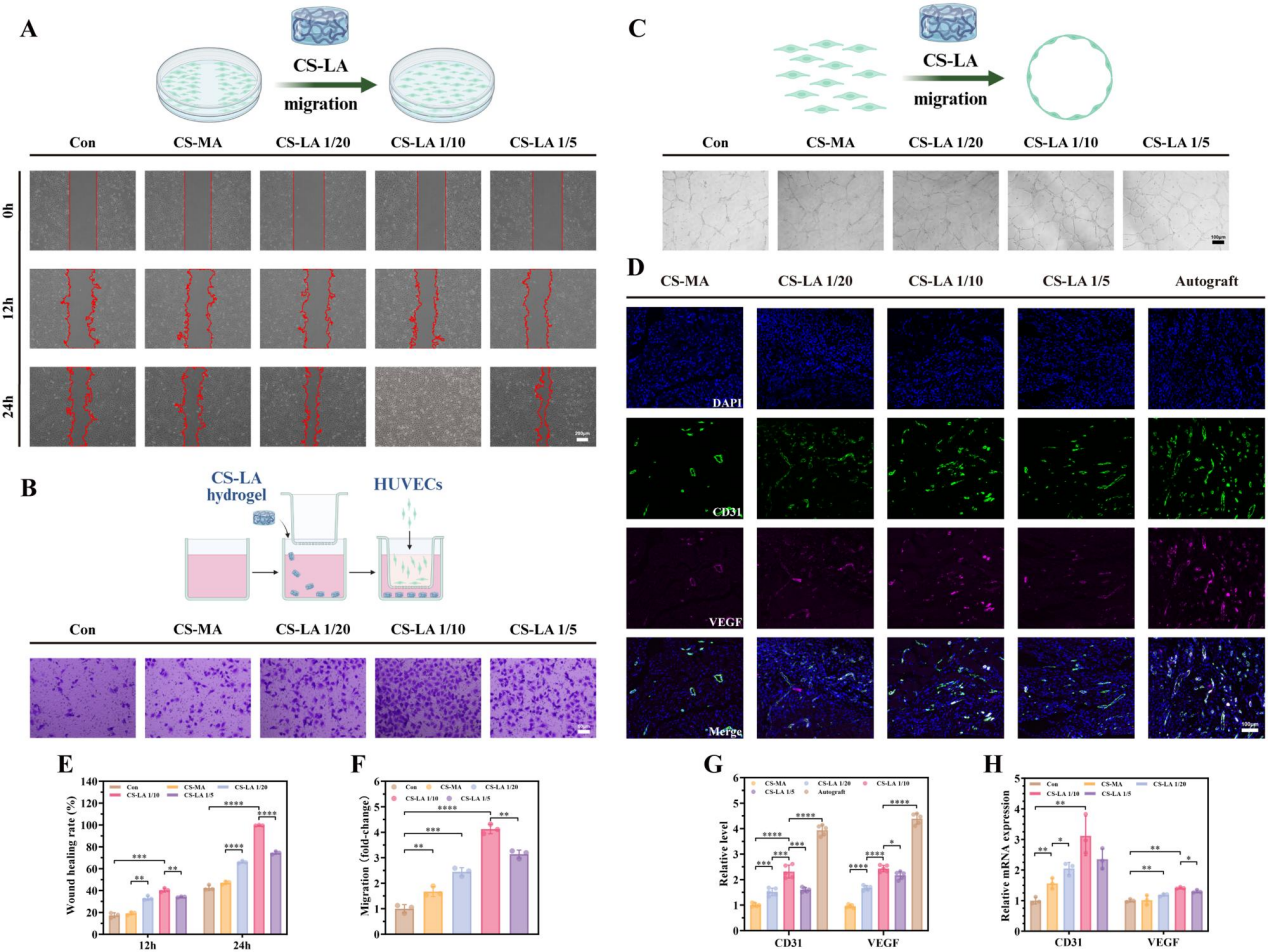
The angiogenic cell effects of CS-LA hydrogel. (**A**) Scratch wound healing tests images from 0 to 24 h accessing HUVEC migration. Scale bar: 200 μm. (**B**) Six-hour tube formation assay images. Scale bar: 100 μm. (**C**) Twenty-four-h transwell method assessing HUVEC migration. Scale bar: 50 μm. (**D**) Immunofluorescence staining of CD31/VEGF on the proximal nerve stumps at first week post-surgery. Scale bar: 100 μm. (**E**) Statistical data of scratch tests (*n* = 3). (**F**) Statistical data on cell migration by transwell assays (*n* = 3). (**G**) Statistical data of CD31 and VEGF fluorescence staining (*n* = 5). (**H**) CD31 and VEGF expression *in vitro* cell experiments obtained through RT-qPCR (*n* = 3). *P < 0.05, **P < 0.01, ***P < 0.001, ****P < 0.0001.

On the other hand, following PNI, the disruption of tissue vasculature impairs the delivery of oxygen, neurotrophic factors, and signaling molecules—significantly inhibiting cell survival and axonal regeneration [34]. Besides, a synergistic relationship exists between blood vessels and peripheral nerves: the nervous system releases growth factors to promote angiogenesis, while newly formed blood vessels secrete growth factors and chemokines to guide directional growth of nerves [35]. Thus, evaluating the effect of CS-LA on cell migration and tube formation—the primary processes of angiogenesis—was necessary. First, we performed CCK-8 assays to assess the effect of CS-LA on HUVECs (Supplementary Figure S6). Compared with the control group, CS-LA 1/10 and CS-LA 1/20 exhibited a significant proliferation-promoting effect at 5 days. Subsequently, the results of scratch experiments (Figure 4A and E), transwell experiments (Figure 4B and F), and tube formation experiments (Figure 4C and Supplementary Figure S7A–C) *in vitro* showed that LA incorporation exerted a positive effect on cell migration, accelerating cell movement and endowing the hydrogel with potential to promote vascular regeneration and axon growth. Additionally, with increasing LA ratio, the angiogenesis effect tended to increase. However, an excessively high LA ratio may impair cell migration behavior, reducing regeneration efficiency. The scratch area tended to further decrease with increasing LA ratio.

The *in vivo* angiogenesis effect of CS-LA/P(MMD-CL) was also evaluated. At first week post-surgery, immunofluorescence staining was performed on the proximal nerve stumps to observe and quantify the protein expression levels of CD31 and VEGF (Figure 4D and G). Although the protein levels in CS-LA groups were lower than those in the autograft groups, they were higher than those in CS-MA groups. Moreover, RT-qPCR was used to detect the expression levels of CD31 and VEGF genes *in vitro*-cultured HUVECs (Figure 4H). The CS-LA groups showed upregulated expression of both CD31 and VEGF, further confirming the angiogenesis-promoting potential of CS-LA. With ROS being scavenged, M2 macrophages and Schwann cells synergistically secrete VEGF-A that activates the PI3K/AKT pathway in vascular endothelial cells, enhancing endothelial cell proliferation, adhesion, and lumen formation to accelerate functional blood vessel reconstruction [7, 36, 37]. This result indicates that CS-LA possesses a certain potential to promote angiogenesis.

### Antioxidant effect of CS-LA

**Figure 5:**
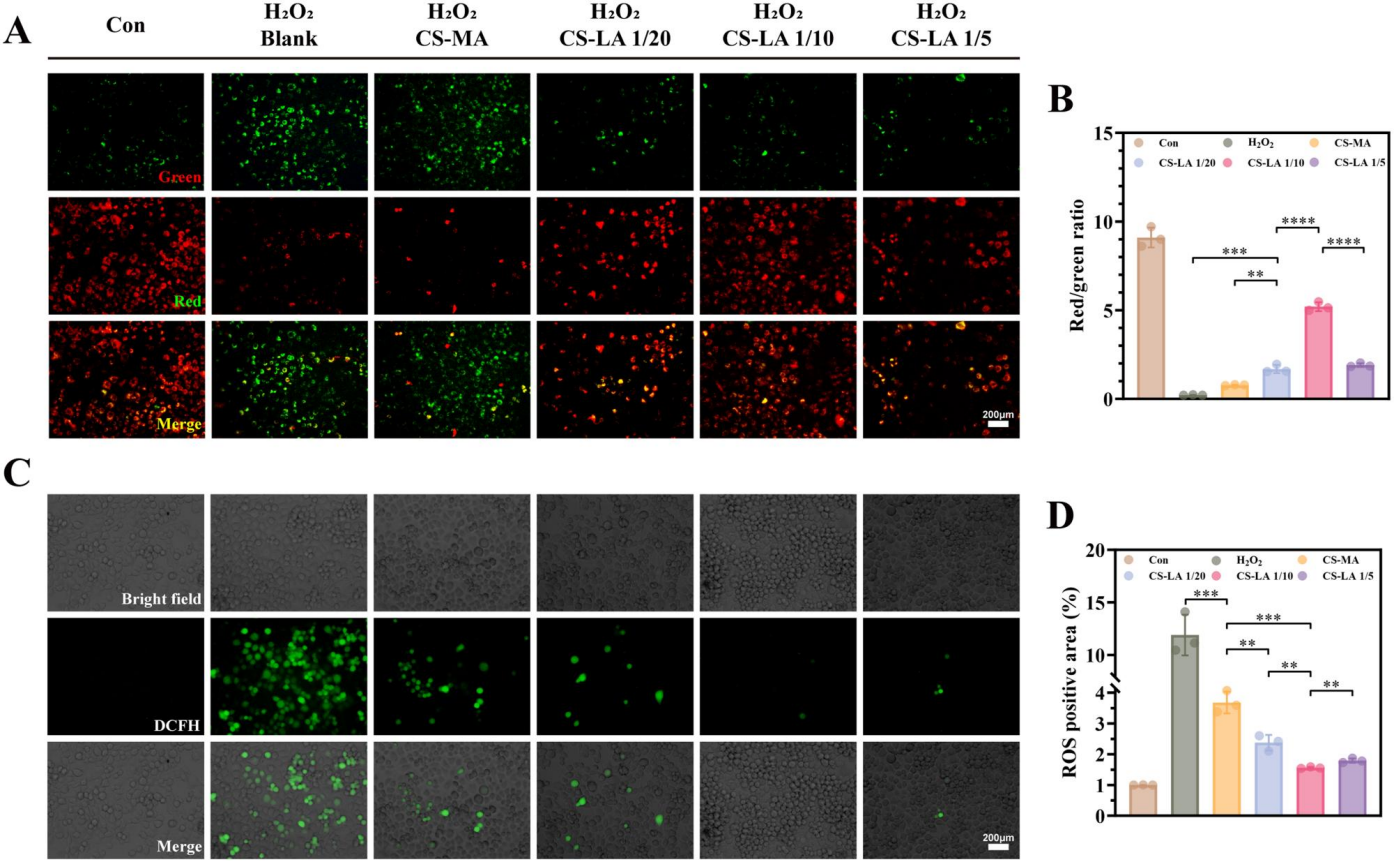
Antioxidative effects of CS-LA hydrogel. (**A**) The MMP of RSC was detected using JC-1 fluorescent probes. Scale bar: 200 μm. (**B**) Statistical data of the red and green fluorescence ration in RSC (*n* = 3). (**C**) The effect of CS-LA hydrogels on ROS in RSCs. Scale bar: 200 μm. (**D**) Statistical data of CS-LA hydrogels antioxidant effect (*n* = 3). *P < 0.05, **P < 0.01, ***P < 0.001, ****P < 0.0001.

In the early stage of PNI, excessive ROS generated by oxidative stress often causes oxidative damage to cells, inhibits the growth of regenerating axons, and induces inflammatory necrosis of adjacent normal cells [4]. Therefore, reducing ROS levels at the injury site is conducive to promoting the nerve regeneration process. The effect of CS-LA on mitochondrial function after oxidative damage was assessed using the JC-1 assay kit. When cells maintain good viability and high MMP, JC-1 aggregates in the mitochondrial matrix that emit red fluorescence. In contrast, JC-1 exists in a monomeric form and emits green fluorescence. Thus, the ratio of red to green fluorescence reflects the functional state of mitochondria. Figure 5A shows that the CS-LA groups exhibited increased MMP levels in cells. Compared with the control groups, the fluorescence ratio of the CS-LA 1/10 group reached 57.2% of that in the control group (Figure 5B). The *in vitro* antioxidant effect of CS-LA hydrogels was further evaluated using the DCFH-DA staining assay (Figure 5C). The CS-LA hydrogel effectively reduced ROS levels in RSCs, with the CS-LA 1/10 group achieving a maximum reduction of 86.9% (Figure 5D). These results further confirm that the CS-LA hydrogels can reduce ROS levels and alleviate mitochondrial oxidative damage.

### Anti-inflammatory capacity of CS-LA

**Figure 6:**
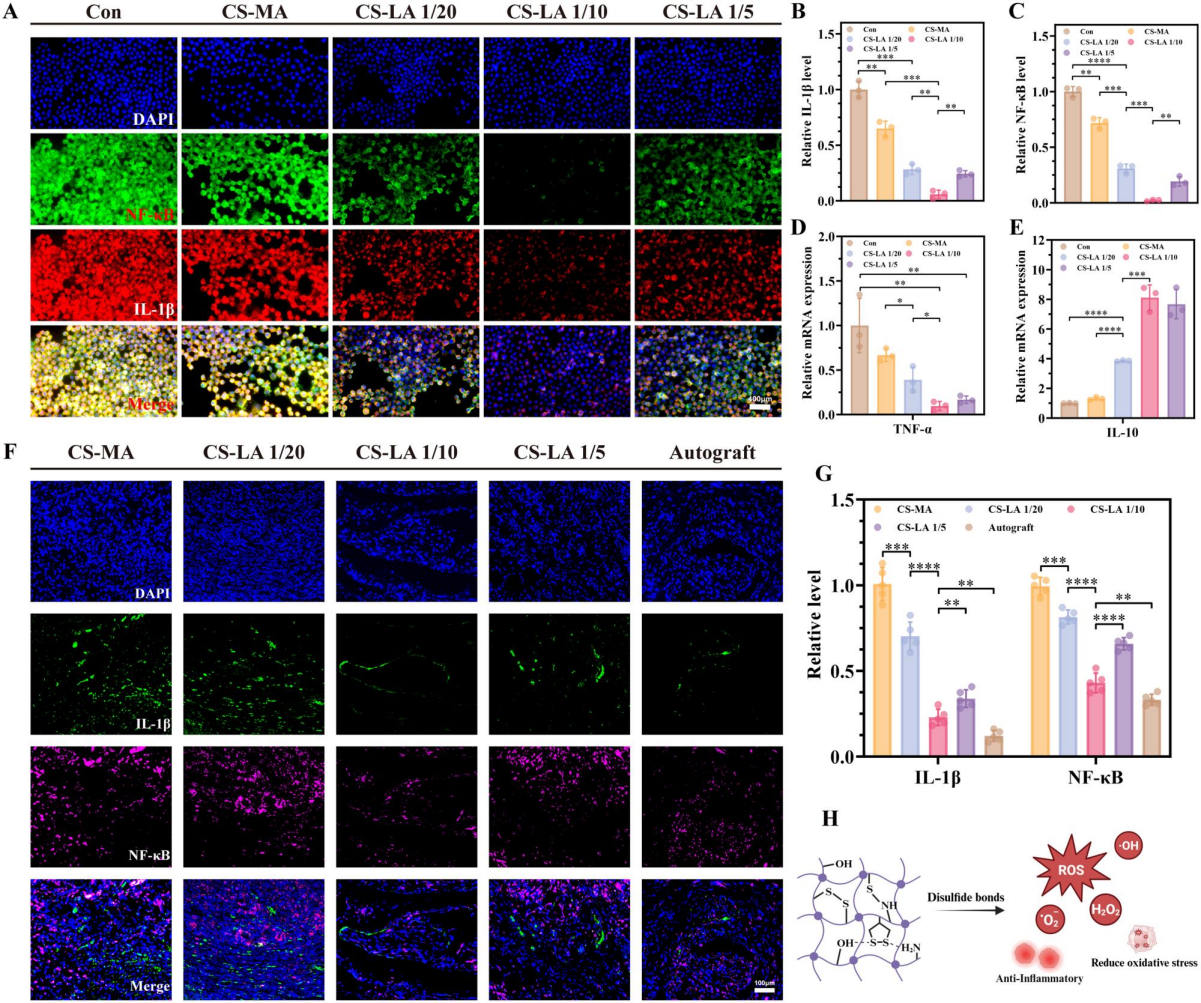
Anti-inflammatory and antioxidative effects of CS-LA hydrogel. (**A**) CS-LA hydrogels reduce inflammatory responses *in vitro*. Scale bar: 400 μm. (**B, C**) Statistical data of IL-1β and NF-κB fluorescence intensity (*n* = 3). (**D, E**) TNF-α and IL-10 expression *in vitro* cell experiments obtained by RT-qPCR (*n* = 3). (**F**) IL-1β/NF-κB immunofluorescence staining on proximal nerve stumps at first week post-surgery. Scale bar: 100 μm. (**G**) Statistical data of IL-1β and NF-κB fluorescence staining (*n* = 5). (**H**) Schematic diagram of CS-LA hydrogel network reacts with ROS reducing oxidative stress of injury microenvironment. *P < 0.05, **P < 0.01, ***P < 0.001, ****P < 0.0001.

After PNI, the neural microenvironment rapidly transitions to an acute inflammatory state and releases oxidative substances, which are initially intended to create a microenvironment conductive to clearing tissue debris and combating pathogens [38]. However, this process is typically excessive, inhibiting tissue regeneration and impeding the outgrowth of regenerating axons. Therefore, we further investigated the effect of CS-LA on the inflammatory factors NF-κB, IL-1β, TNF-α and IL-10 using RAW264.7 cells and RT-qPCR. Immunofluorescence results (Figure 6A) and statistical analyses (Figure 6B and C) showed that CS-LA significantly reduced the expression of NF-κB and IL-1β, with the relative fluorescence intensities being only 0.02 and 0.06 times those of the control group, respectively. Additionally, CS-LA downregulated the gene expression of TNF-α and upregulated the gene expression of IL-10 (Figure 6D and E). These findings indicate that CS-LA can mitigate the inflammatory response, thereby facilitating the recovery of injured nerves.

We also evaluated the *in vivo* anti-inflammatory effect of CS-LA/P(MMD-CL) by performing immunofluorescence staining of the proximal nerve stumps at first week post-surgery (Figure 6F). Results showed that the expression levels of NF-κB and IL-1β in the CS-LA groups were decreased, and the expression levels in the CS-LA 1/10 groups were close to those in the Autograft groups (Figure 6G). This confirms that after nerve injury, CS-LA/P(MMD-CL) effectively reduced the expression of inflammation-related factors and demonstrated a favorable anti-inflammatory capacity (Figure 6H).

### CS-LA/P(MMD-CL) promotes nerve regeneration

**Figure 7:**
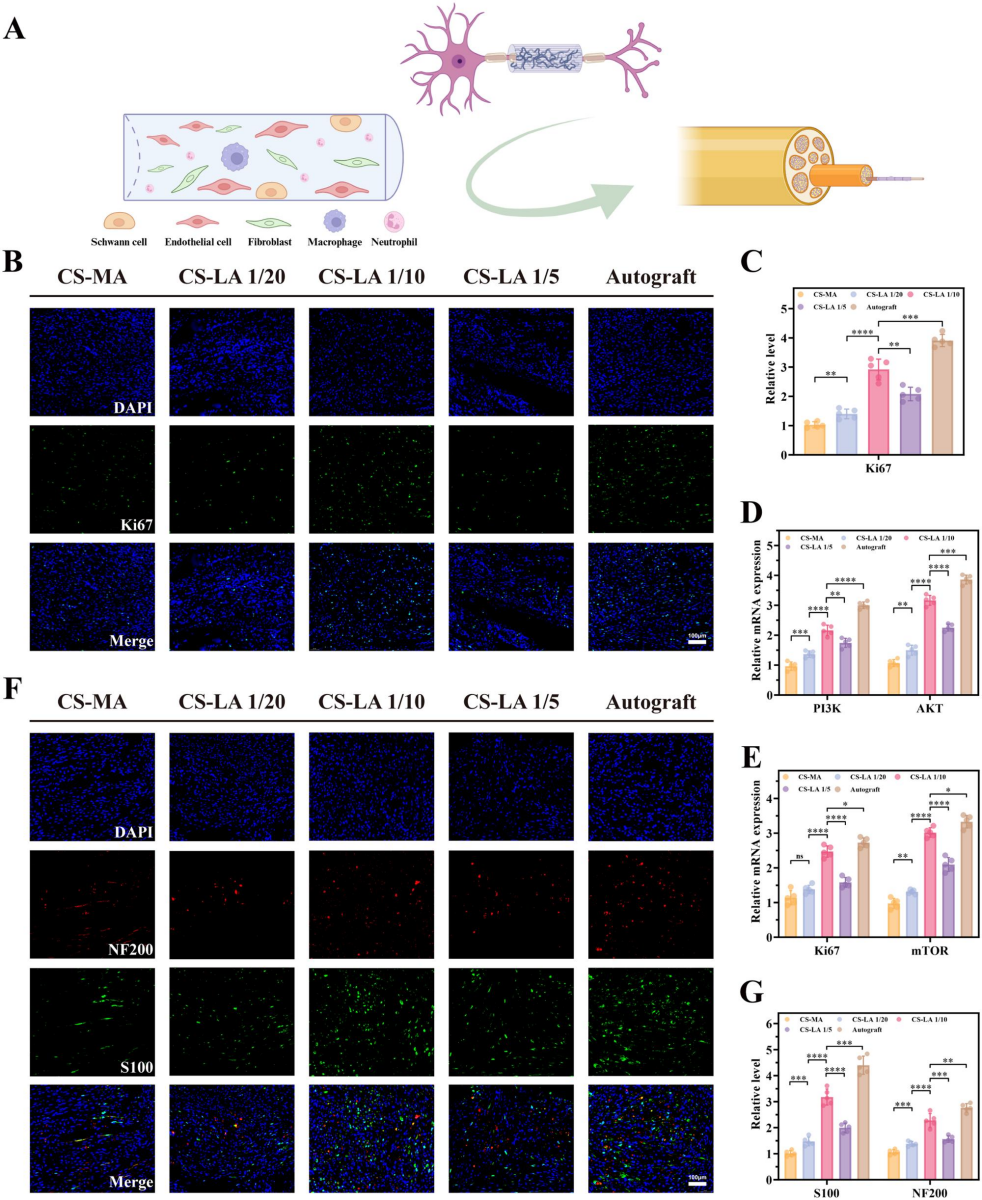
The proliferative effects of CS-LA hydrogel. (**A**) Schematic diagram of CS-LA/P(MMD-CL) promotes regenerated axon growth. (**B**) Immunofluorescence staining of Ki67 on the proximal nerve stumps at the first week post-surgery. Scale bar: 100 μm. (**C**) Statistical data of Ki67 fluorescence intensity (*n* = 5). (**D**) PI3K and AKT expression at the first week post-surgery were obtained by RT-qPCR (*n* = 5). (**E**) Ki67 and mTOR expression at the first week post-surgery were obtained by RT-qPCR (*n* = 5). (**F**) Immunofluorescence staining of NF200/S100 on the proximal nerve stumps at first week post-surgery. Scale bar: 100 μm. (**G**) Statistical data of NF200 and S100 fluorescence staining (*n* = 5). *P < 0.05, **P < 0.01, ***P < 0.001, ****P < 0.0001.

Cell proliferation is an essential process for peripheral nerve regeneration. Excessive inflammation and oxidative stress can inhibit cell proliferation, whereas a favorable tissue regeneration microenvironment is typically accompanied by efficient cell proliferation (Figure 7A). Ki67, a characteristic protein of cell mitosis, was analyzed via immunofluorescence staining in the proximal nerve stumps (Figure 7B and C) and gene expression detection (Figure 7E). The results showed that CS-LA/P(MMD-CL) effectively enhanced cell proliferation. The PI3K/AKT/mTOR signaling pathway has been well documented to play a crucial role in cell proliferation [39, 40]. Therefore, we focused on exploring the relationship between ROS scavenging and the activation of this pathway. We performed western blotting assays in H_2_O_2_-treated RSCs to elucidate the mechanism. As shown in the representative blots (Supplementary Figure S8), compared to the control group, H_2_O_2_ treatment significantly reduced the phosphorylation levels of PI3K, AKT and mTOR, indicating that oxidative stress suppresses the activity of this pathway. Importantly, treatment with CS-LA 1/10 effectively reversed this inhibition, restoring the phosphorylation of PI3K, AKT and mTOR. In contrast, the addition of the PI3K/AKT inhibitor LY294002 completely abolished the CS-LA-mediated restoration of p-AKT and p-mTOR levels, demonstrating that the activation of the PI3K/AKT/mTOR pathway by CS-LA under oxidative stress is dependent on the upstream kinase activity of PI3K. RT-qPCR analysis revealed that CS-LA/P(MMD-CL) upregulated the expression levels of PI3K, AKT (Figure 7D), and mTOR (Figure 7E). What is noteworthy is that the expression level of CS-LA 1/10 was the closest to those in the Autograft groups. Moreover, we performed western blot analysis to detect the protein levels of key components and their phosphorylation forms (Supplementary Figure S9). As shown in representative blots, CS-LA/P(MMD-CL) significantly increased the phosphorylation levels of PI3K, AKT and mTOR. In contrast, total protein levels of PI3K, AKT and mTOR remained relatively stable across all groups. These findings were consistent with our RT-qPCR results. Together, these results strongly indicate that CS-LA/P(MMD-CL) exerts its ROS-scavenging and cytoprotective effects, which in turn activate the PI3K/AKT/mTOR signaling cascade to promote cell proliferation under oxidative stress conditions.

To assess the nerve regeneration-promoting capacity, immunofluorescence staining for NF200/S100 was conducted on the proximal nerve stumps at first week post-surgery (Figure 7F), followed by statistical analysis (Figure 7G). The expression levels of NF200 and S100 were increased in the CS-LA groups. Although the expression level in CS-LA 1/10 groups was close to that in Autograft groups, CS-LA 1/5 groups showed a poor enhancement effect. Combined with the results of *in vitro* cell experiments, this phenomenon could be attributed to the fact that an excessively high concentration of lipoic acid would inhibit the biological activity of cells.

### CS-LA/P(MMD-CL) improves the morphology and structure of repaired nerves

**Figure 8:**
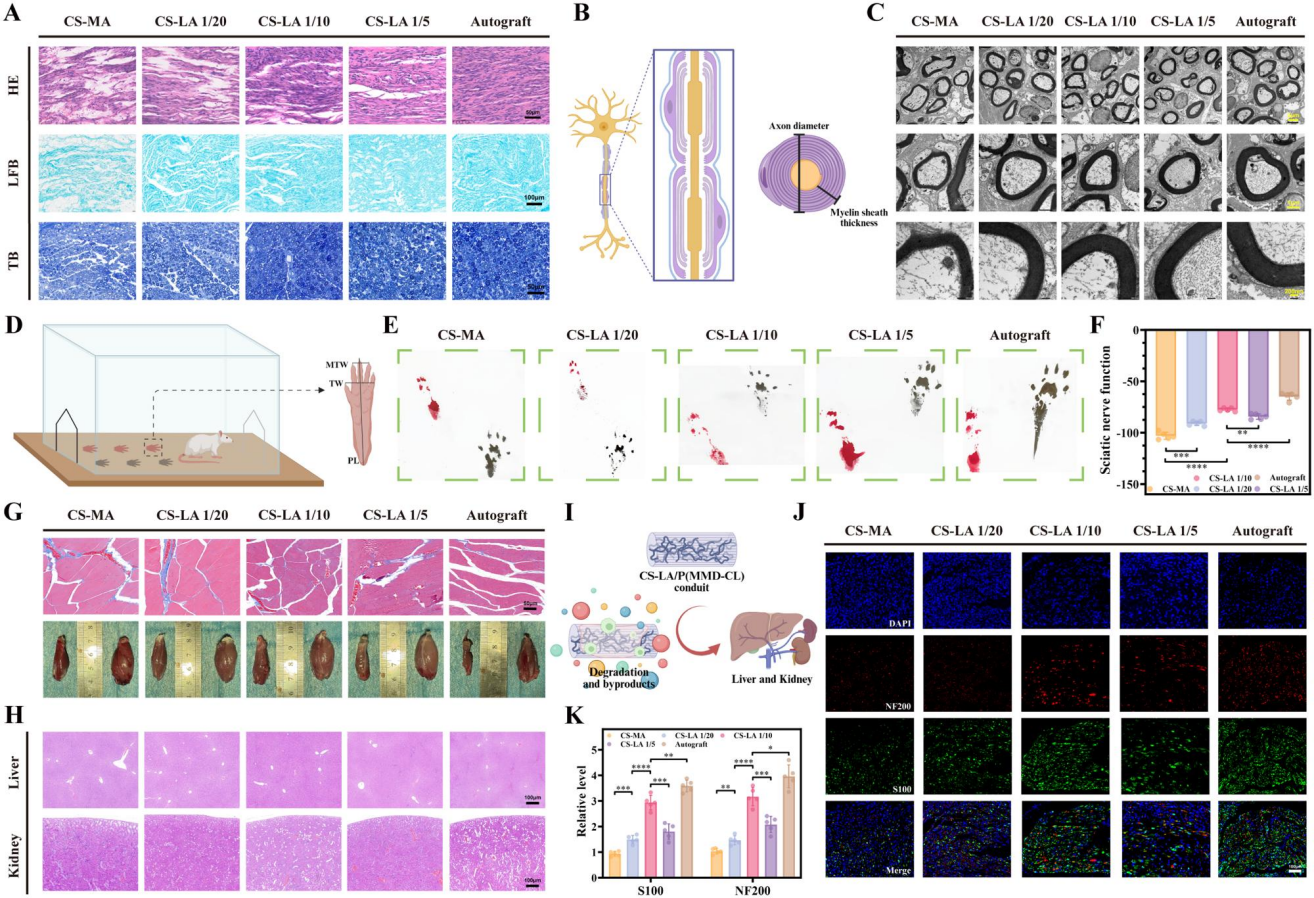
The axonal morphology and nerve function recovery effect of CS-LA/P(MMD-CL). (**A**) Stained images of HE, LFB and TB. Scale bars: 50, 100 and 50 μm. (**B**) Images of the structure of mature peripheral nerve. (**C**) TEM images of transverse sections of regenerated nerves were used to observe the structure of myelin sheaths. Scale bars: 2, 1 and 200 nm. (**D**) Typical image of the rat footprints collection device. (**E**) Schematic diagram of rat footprints (normal side: black; injured side: red). (**F**) SFI values of rats in the 12th week (*n* = 5). (**G**) Muscle contrast images (left: injured side; right: normal side) and Masson staining of the injured side at 12th week post-surgery. Scale bar: 50 μm. (**H**) H&E staining of the liver and kidney at the 12th week post-surgery. Scale bar: 100 μm. (**I**) Schematic diagram of metabolic pathways of degradation by-products of CS-LA/P(MMD-CL). (**J**) Immunofluorescence staining of NF200/S100 on longitudinal sections of the proximal nerve stumps at 12th week post-surgery. Scale bar: 100 μm. (**K**) Statistical data of NF200 and S100 fluorescence staining (*n* = 5). *P < 0.05, **P < 0.01, ***P < 0.001, ****P < 0.0001.

To further assess SD rats’ recovery at 12th weeks post-surgery, HE, LFB and TB staining were performed on nerve sections (Figure 8A). HE staining results showed that the number of Schwann cells in CS-LA groups was between that in CS-MA groups and the Autograft groups (Supplementary Figure S11A), with the Schwann cell count in the CS-LA 1/10 groups being close to that in the autograft groups. LFB, TB and TEM (Figure 8C) were used to observe the density and morphology of regenerated axons. A schematic diagram (Figure 8B) illustrated the mature peripheral nerve structure. Myelination was observed in the regenerated tissues of all five groups. Further quantitative analysis revealed that the myelin density (Supplementary Figure S11B), axon diameter (Supplementary Figure S11C), and myelin sheath thickness (Supplementary Figure S11D) of the regenerated nerve tissues followed a pattern similar to that of the Schwann cell count. These findings indicate that CS-LA/P(MMD-CL) can improve the repair level of regenerated nerves, with the highest improvement observed when the molar ratio of CS to LA was 1/10. This result is consistent with the findings of *in vitro* experiments. Meanwhile, immunofluorescence staining for NF200/S100 on longitudinal sections (Figure 8J) and for NF200 on cross-sections (Supplementary Figure S12A) was performed on proximal nerve stumps at 12 weeks post-surgery. Quantitative analysis (Figure 8K and Supplementary Figure S12B) of the results showed that the fluorescence intensity in the CS-LA 1/10 group was relatively close to that in the autograft group, which further supports the above conclusion.

### CS-LA/P(MMD-CL) promotes nerve function recovery

Walking footprints of SD rats were collected at the 12th week post-surgery for evaluating the effect of CS-LA/P(MMD-CL) on peripheral nerve function recovery (Figure 8D and E), and the sciatic functional index (SFI) was calculated via measurement and analysis. An SFI value of 0 indicates intact peripheral nerve function, and a higher SFI value reflects nerve function closer to the normal state. The SFI values of the CS-MA, CS-LA 1/20, CS-LA 1/10, CS-LA 1/5 and autograft groups were −103.05 ± 3.46, −91.13 ± 2.01, −77.51 ± 1.51, −84.34 ± 2.64 and −64.81 ± 3.59, respectively (Figure 8F). These results demonstrate that CS-LA/P(MMD-CL) exerts a promoting effect on peripheral nerve function recovery. Masson staining was performed on the gastrocnemius muscle innervated by the injured nerve to observe the morphology of muscle fibers (stained red) and collagen deposition (stained blue) (Figure 8G), and statistical analysis of collagen deposition was conducted (Supplementary Figure S11E). The results showed that the muscle fibers in the CS-LA groups were relatively more ordered, with less collagen deposition. Meanwhile, the gastrocnemius muscles on both sides were weighed, and the degree of gastrocnemius atrophy was evaluated by the ratio of the weight of the surgical side (left side) to that of the normal side (right side) (Supplementary Figure S11F). The ratios for the CS-LA groups were 23.5%, 36.6% and 33.0%, respectively, while the ratio for the autograft group was 60.9%. These findings indicate that CS-LA/P(MMD-CL) alleviates gastrocnemius atrophy to a certain extent. Furthermore, to evaluate the biocompatibility of CS-LA/P(MMD-CL) degradation byproducts (Figure 8I), H&E staining was performed on heart, liver, spleen and kidney sections at 12 weeks post-surgery, and no obvious histological damage or adverse effects were observed (Figure 8H and Supplementary Figure S13). Collectively, the above results confirm that CS-LA/P(MMD-CL) plays a positive role in promoting the repair of peripheral nerve function with great biocompatibility.

## Conclusion

In summary, to address the oxidative stress and excessive tissue inflammation after PNI, we designed CS-LA to fabricate an ROS-scavenging nerve conduit, CS-LA/P(MMD-CL). This system leverages the cleavage and reconstruction of S-S to achieve gelation without introducing photoinitiators, while avoiding significantly prolonged gelation time. Notably, the high reactivity of S-S toward ROS alleviates oxidative stress and modulates the expression level of inflammatory factors, thereby exerting anti-inflammatory effects to heal excessive inflammation. Furthermore, our findings demonstrate that the modulation of excessive inflammation exerts a positive promotional effect on subsequent cell proliferation and angiogenesis. This modulation provides sufficient nutrients for regenerating axons, promotes axon regeneration, effectively enhances nerve function recovery and reduces target organ atrophy. Additionally, the CS-LA/P(MMD-CL) system exhibits favorable biocompatibility, with degradation byproducts exerting no significant adverse effects on major organs, such as the heart, liver, spleen, and kidneys. Beyond PNI repair, the core design of CS-LA hydrogel—targeting oxidative stress and excessive inflammation via ROS-responsive S-S and rapid photocrosslinking under 365 nm UV light, which allowing for *in situ* gelation that conforms to the irregular shape of injury sites—holds broad biomedical application potential, as these two pathological hallmarks are common in numerous diseases, such as diabetic wound healing, cancer, hypoxic or infected wounds and so on. All in all, this study provides a novel strategy for modulating excessive inflammation in the PNI microenvironment and offers experimental evidence to support the ability of CS-LA hydrogels in promoting nerve regeneration.

## Supplementary Material

rbag068_Supplementary_Data
