# Supplementary material for: ROS-scavenging lipoic acid-modified chitosan hydrogels with rapid photocrosslinked ability accelerates peripheral nerve regeneration
Source: Regen Biomater. 2026 Apr 3;13:rbag068. doi: 10.1093/rb/rbag068 (PMC13110000; doi:10.1093/rb/rbag068)
Supplement: rbag068_Supplementary_Data [file rbag068_supplementary_data.zip › Supplementary_data.docx]

**Supplementary Data**

**ROS-scavenging lipoic acid-modified chitosan hydrogels with rapid photocrosslinked ability accelerates peripheral nerve regeneration**

*Xianglong Chen^a,b#^, Ren Gao^c,d#^, Xinyue Liang^a,b^, Kun Liu^a^, Zhong Wei^d^, Xiaopei Wu^a^, Takashi Goto ^a^, Chengjie Xiong^c,d*^, Feng Xu^c,d*^ , Honglian Dai^a,b*^*

*^a^State Key Laboratory of Advanced Technology for Materials Synthesis and Processing, and Hubei Key Discipline Laboratory of Orthopedic Tissue Injury and Repair, Wuhan University of Technology, Wuhan 430070, China*

*^b^Wuhan University of Technology Advanced Engineering Technology Research Institute of Zhongshan City, Zhongshan 528400, China*

*^c^The First School of Clinical Medicine, Southern Medical University, Guangzhou 510515, China*

*^d^Department of Orthopaedics,* *General Hospital of Central Theater Command, Wuhan 430070, China*

^*^Corresponding authors.

E-mail address: [daihonglian@whut.edu.cn](mailto:daihonglian@whut.edu.cn) (H. Dai), rocket1110@126.com (C. Xiong), fengxu1969@163.com (F. Xu)

^#^These two authors contributed equally to this work.

**Preparation of CS-LA/P(MMD-CL) conduit**

Briefly, 1 g of P(MMD-CL) was dissolved in 6.67 ml of 1,1,1,3,3,3-hexafluoro-2-propanol and stirred overnight to form a 15% (w/v) solution. The solution was loaded into a 10 mL syringe (equipped with a 22-gauge stainless steel needle) and electrospun using an electrospinning spinner device (Ucalery, Beijing) at a flow rate of 1 ml h^-1^ and positive high voltage of 8 kV. Oriented electrospun fibers (2400 rpm) were collected using a rotating drum covered with silicone oil paper. The resulting fibrous membrane was vacuum-dried at room temperature for 24 h to remove the residual solvent. The oriented fiber membrane was cut into a specific size and rolled along the long axis of a Teflon rod to form a conduit with a diameter of 1.5 mm. The fiber conduit was uniformly heated to enhance its compressive strength and finally cut into oriented fiber conduits with a length of approximately 12 mm. All the dimensional parameters of the conduits were rationally designed to match the average transverse diameter of the sciatic nerve and the established sciatic nerve injury model in adult SD rats. Subsequently, the lyophilized CS-LA with different ratios were separately dissolved in deionized water to prepare 4% (w/w) CS-LA solutions. Each solution was injected into lumen of P(MMD-CL) conduits, followed by irradiation with 365 nm ultraviolet light (10 mW cm^-2^) until gelation. The CS-MA/P(MMD-CL) conduits used as the control group were fabricated using an identical procedure.

**Cell culture**

Rat Schwann cells (RSCs) and human umbilical vein endothelial cells (HUVECs) were maintained in DMEM medium supplemented with 10% fetal bovine serum (FBS, SORFA, Beijing, China) and 1% penicillin-streptomycin solution (PS, Vivacell, Shanghai, China), and cultured at 37°C in a humidified atmosphere containing 5% CO_2_.

Composite hydrogels were soaked in 75% ethanol solution for 2 h and then washed three times with PBS solution. Subsequently, hydrogel samples were immersed in DMEM medium (Gibco, USA) at a ratio of 0.1 g mL^-1^, followed by incubation in a constant-temperature shaking chamber for 24 h. Finally, the extracts were filtered through a 0.22 μm bacterial-retention filter and stored at 4°C for further use. For experimental use, 10% FBS (SORFA, Beijing, China) and 1% PS (Vivacell, Shanghai, China) were added to different hydrogel extracts to prepare complete hydrogel extract media.

**RT-qPCR**

To investigate the effect of CS-LA hydrogel on the gene expressions of CD31 and VEGF in HUVECs as well as TNF-α and IL-10 in RAW 264.7 cells, HUVECs and RAW 264.7 cells were seeded in plates at a density of 1 × 10^6^ cells per plate. The cells were cultured in an incubator for 24 h, followed by two washes with PBS. Subsequently, the cells were cultured in DMEM medium containing lipopolysaccharide (LPS, 500 ng mL^-1^) for 12 h. After that, the cells were washed twice with PBS, and the supernatant was replaced with complete medium containing different hydrogel extracts. Next, the cells were co-cultured with the drug for 2 days. The cells were then washed twice with PBS, collected by centrifugation, and gently mixed with 800-1000 μL of Trizol (G3013, Servicebio). Total mRNA was extracted in accordance with the manufacturer's protocol. After reverse transcription and amplification, the concentrations of target genes were quantified using the CFX Connect Real-Time PCR system (Bio-Rad, USA), and the gene expression levels were calculated by the 2^-ΔΔCT^ method. The sequences of the primers used are provided in Supplementary Table S1.

Sciatic nerve specimens were collected from each group at 1st week post-surgery. According to the manufacturer’s protocol, total RNA was extracted from sample using TRIpure reagent (Aidlab Biotechnologies Co., ltd, Beijing). After [reverse transcription](https://www.sciencedirect.com/topics/medicine-and-dentistry/reverse-transcription) and amplification, real-time PCR Stepone plus (ABI, USA) was used to quantify the concentration of target genes. Adopting 2^−ΔΔCT^ method, calculate gene expression levels. The primer sequences used are shown in Supplementary Table S2.

**Western blot**

RSCs were seeded in six-well plates at 1×10^6^ per well and treated for 48 hours. Cells were collected by centrifugation, and 150 μL of radioimmunoprecipitation assay (RIPA) lysate containing 1 mM phenylmethylsulfonyl fluoride was added, and homogenized for 45 minutes at 4°C until cells are fully lysed. Solubilized protein was collected by centrifugation at 12,000*g*, and the supernatant was quantified for protein concentration using bicinchoninic acid (BCA) reagents. Polyacrylamide gels were separated protein samples and were transferred to polyvinylidene fluoride membranes (Sigma-Aldrich, USA). Nonspecific proteins on membranes were blocked with 5% nonfat dry milk at r.t. for 2 hours and then incubated with P-AKT (1:1000, rabbit polyclonal antibody, GB150002, Servicebio), AKT (1:1000, rabbit polyclonal antibody, GB15689, Servicebio), P-PI3K (1:1000, rabbit polyclonal antibody, AF3242, Affinity), PI3K (1:1000, rabbit polyclonal antibody, GB11525, Servicebio), P-mTOR (1:1000, rabbit polyclonal antibody, GB114489, Servicebio), mTOR (1:1000, rabbit polyclonal antibody, GB111840, Servicebio) overnight at 4°C. Membranes were probed with the corresponding secondary antibody (matched with primary antibody species), then imaged with a chemiluminescence imager according to the present program, and the scanned original images were analyzed using AIWBwell^TM^ (Servicebio) analysis software.

For animal experiments, Nerve-regenerated segments were harvested at 1st week post-surgery and cut into fine tissue fragments. RIPA lysate was added at a ratio of 200 μL per 20 mg of tissue. After lysis, the samples were centrifuged at 12,000*g* for 5 minutes to collect the supernatant containing total protein, followed by Western blot analysis performed as previously described.

**The repair of traumatic PNI of SD rats**

Preparation of CS-LA/P(MMD-CL) conduit: The freeze-dried CS-LA was sterilized and then dissolved in sterile PBS to prepare a 4% (w/w) CS-LA hydrogel precursor solution. Subsequently, 100 μL of the precursor solution was injected into the sterilized hollow P(MMD-CL) conduit, which was immediately irradiated with 365 nm UV light (10 mW·cm^-2^) until gelated, yielding the CS-LA/P(MMD-CL) peripheral nerve conduit.

Animal surgery: SD rats were anesthetized by intraperitoneal injection of 10% chloral hydrate solution (400 mg/kg) and fixed in the prone position on a surgical table. An incision was made on the hind limb, and the skin and subcutaneous tissue were dissected layer by layer to expose the sciatic nerve. A 10‑mm segment of the nerve trunk was excised to create a 10‑mm sciatic nerve defect. The nerve conduits or autologous nerve grafts were sutured to the proximal and distal stumps of the injured nerve using 8‑0 PLGA sutures. The muscle layer and skin were closed with 4‑0 PLGA sutures (Fig. S10). Postoperatively, the incision was disinfected with iodophor, and the rats were housed individually with free access to food and water.


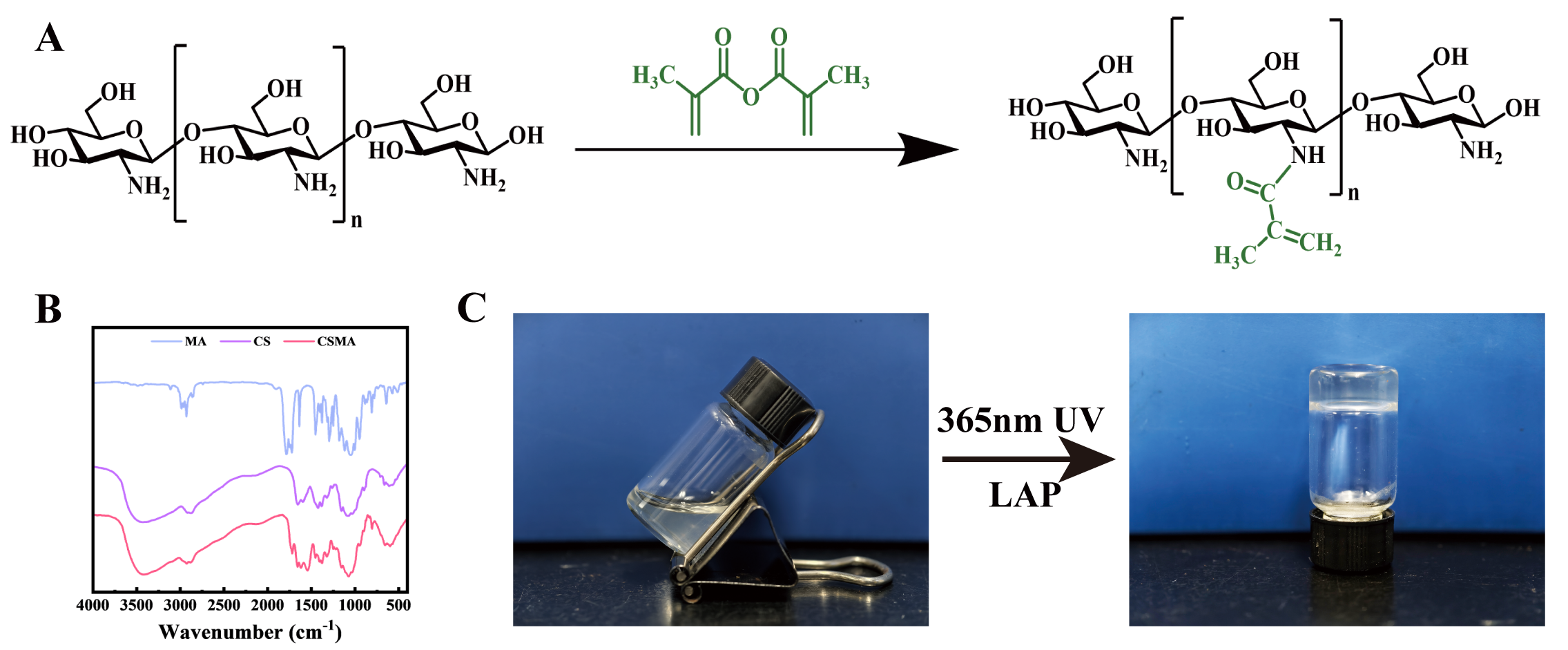


**Figure S1.** (A) Synthesis process of CSMA. (B) FT-IR spectra of CS, MA, and CSMA powder. (C) The formation of CSMA hydrogel under irradiation of 365 nm UV light.


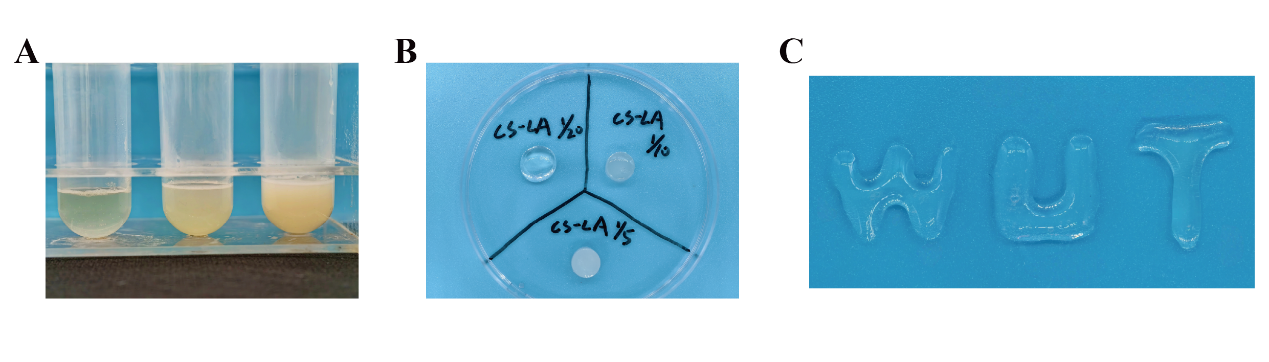


**Figure S2.** (A) Digital images of different CS-LA aqueous solution. (B) Digital images of different CS-LA hydrogel. (C) WUT writing of CS-LA hydrogel.


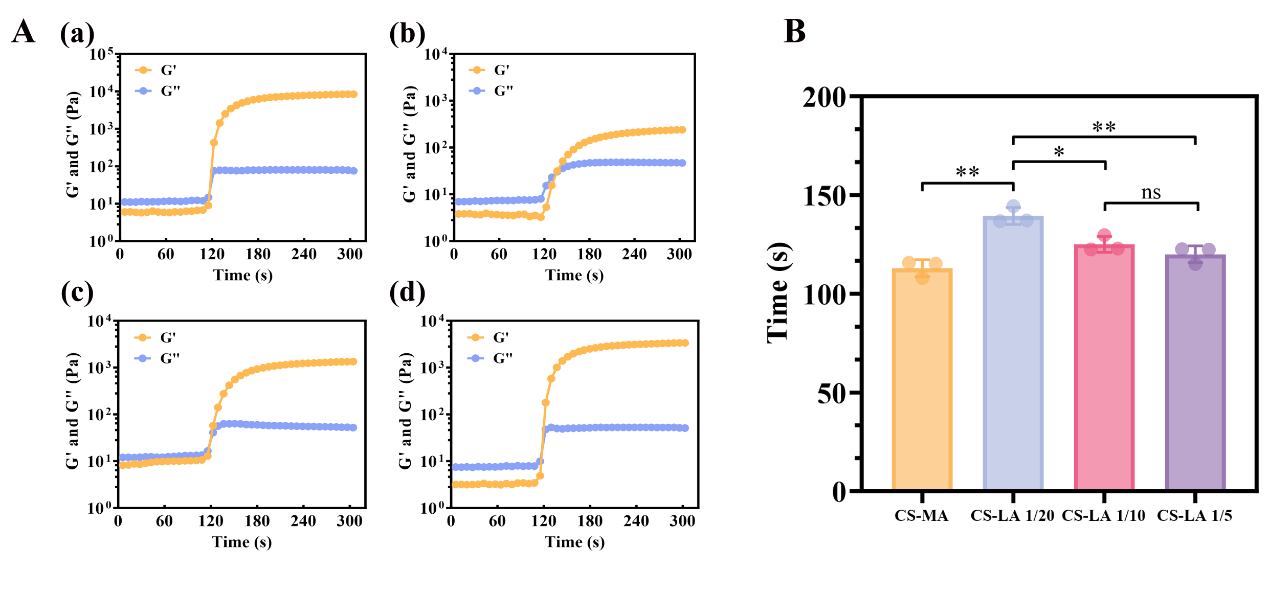


**Figure S3.** (A) Rotational rheological properties of (a) CS-MA, (b) CS-LA 1/20, (c) CS-LA 1/10, and (d) CS-LA 1/5 hydrogels (storage modulus G’ and loss modulus G’’). (B) statistics of gelation time through rotational rheological properties (when storage modulus surpasses loss modulus, G’ > G’’). **P* < 0.05, ***P* < 0.01, ****P* < 0.001, *****P* < 0.0001.


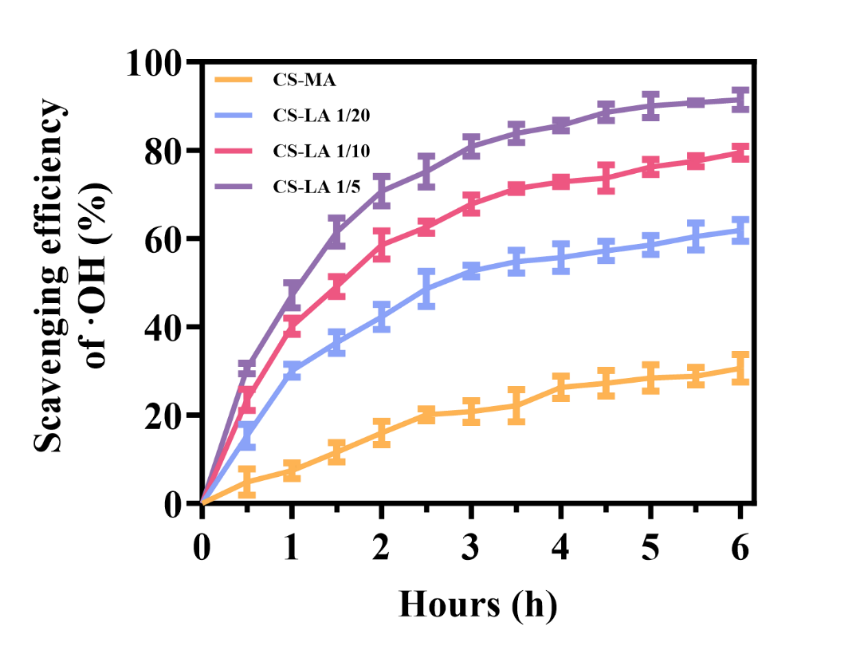


**Figure S4.** Scavenging efficiency of ·OH of CS-LA and CS-MA hydrogels (n = 3).


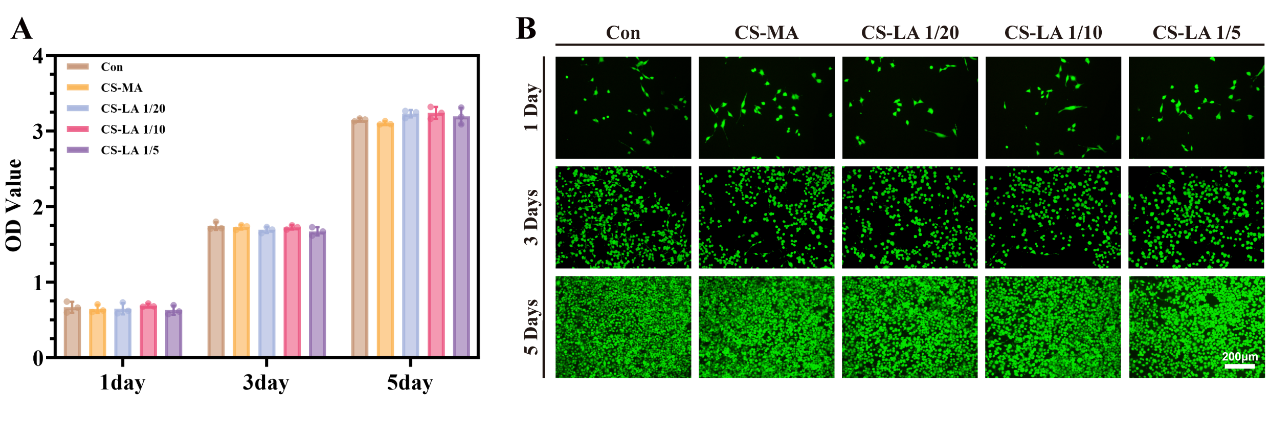


**Figure S5.** (A) The effect of CS-MA and different CS-LA hydrogels on the activity of RSCs. (B) LIVE/DEAD staining of RSCs cultured with CS-MA and CS-LA hydrogels for 1, 3, and 5 days. Scale bar: 200 μm.


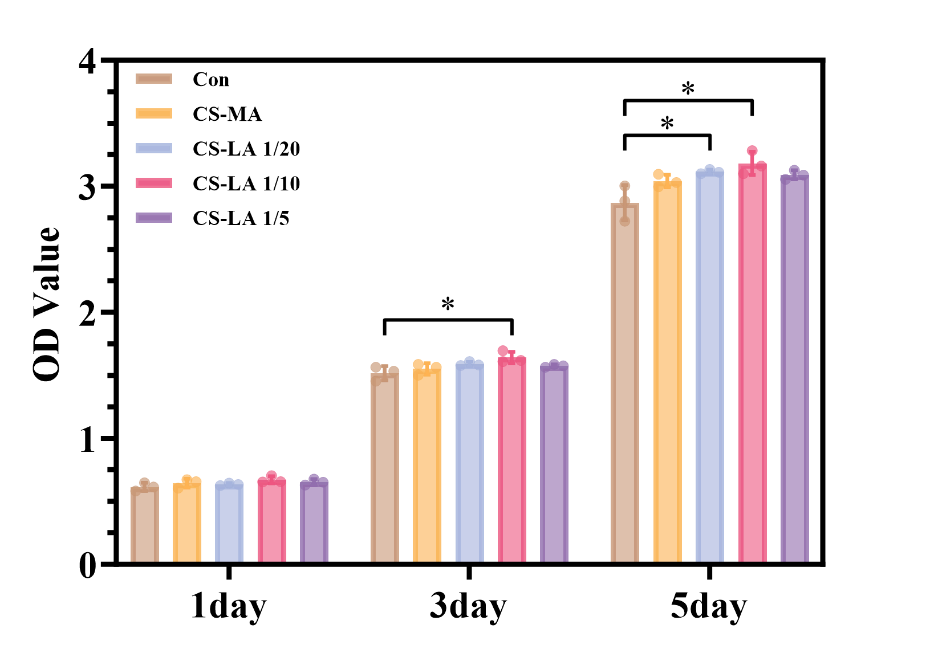


**Figure S6.** The effect of CS-MA and different CS-LA hydrogels on the activity of HUVECs. **P* < 0.05, ***P* < 0.01, ****P* < 0.001, *****P* < 0.0001.


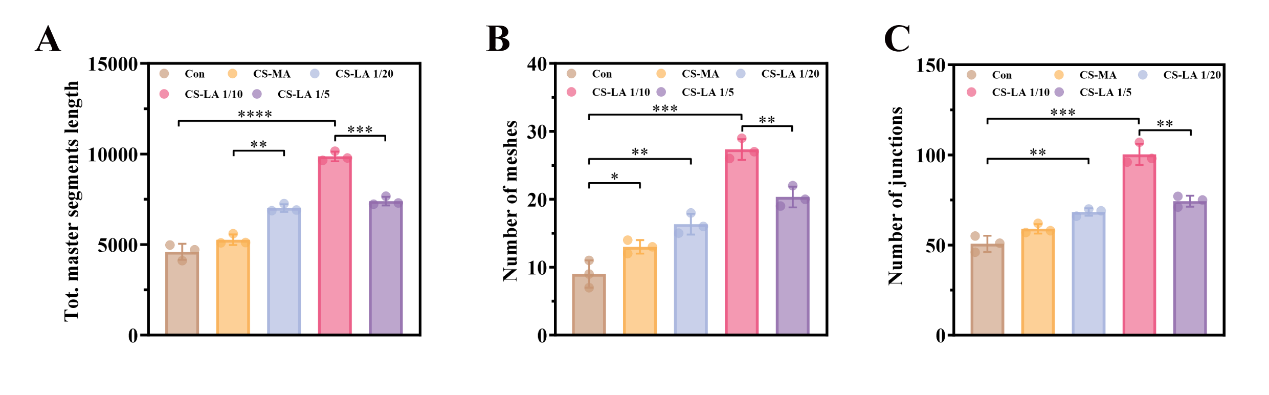


**Figure S7.** (A, B and C) Summary data of tube formation assays (n = 3). **P* < 0.05, ***P* < 0.01, ****P* < 0.001, *****P* < 0.0001.


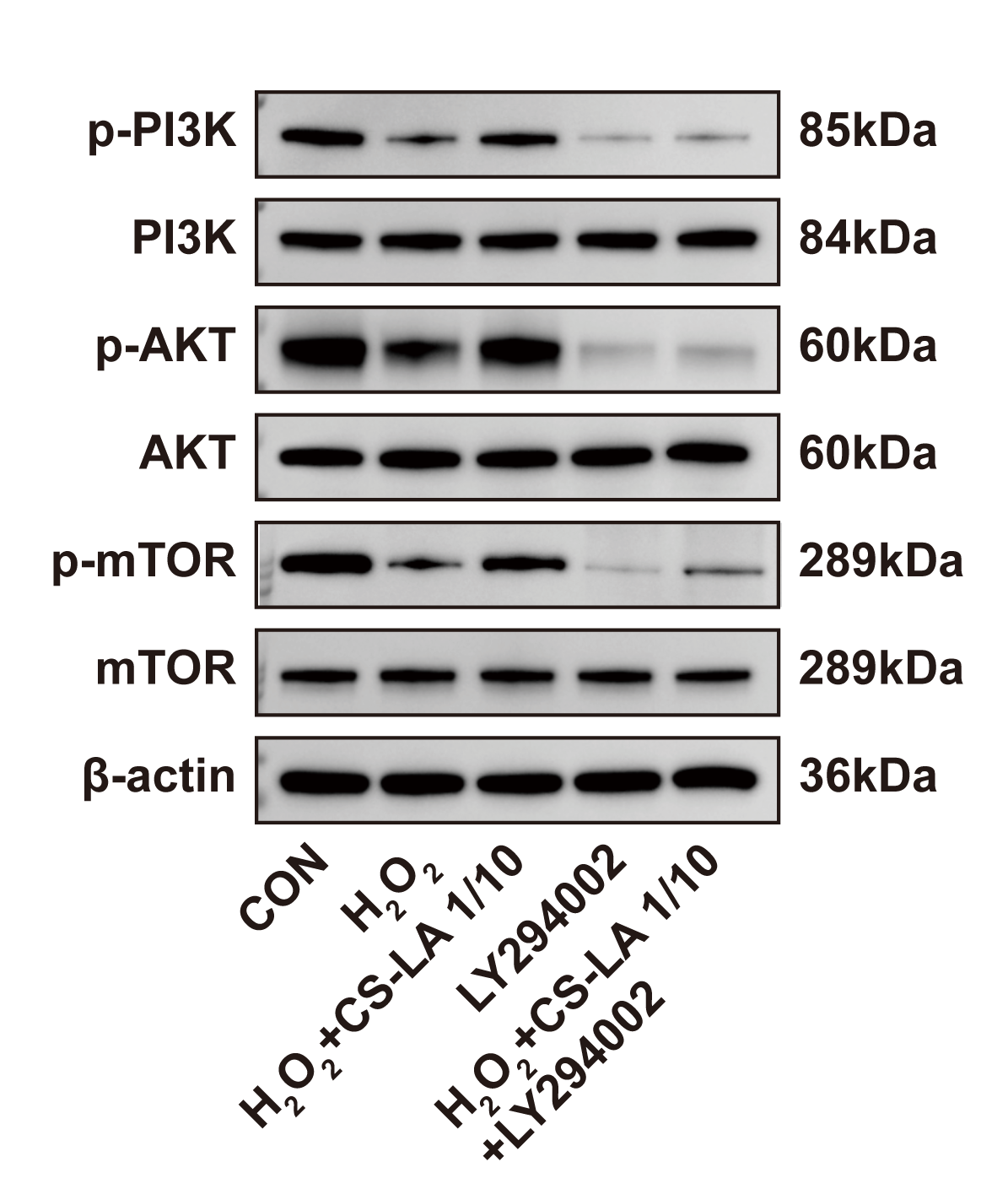


**Figure S8.** Western blot results of protein expression of p-PI3K, PI3K, p-AKT, AKT, p-mTOR and mTOR in RSCs from CON, H_2_O_2_, H_2_O_2_ + CS-LA 1/10, LY294002, H_2_O_2_ + CS-LA 1/10 + LY294002 groups.


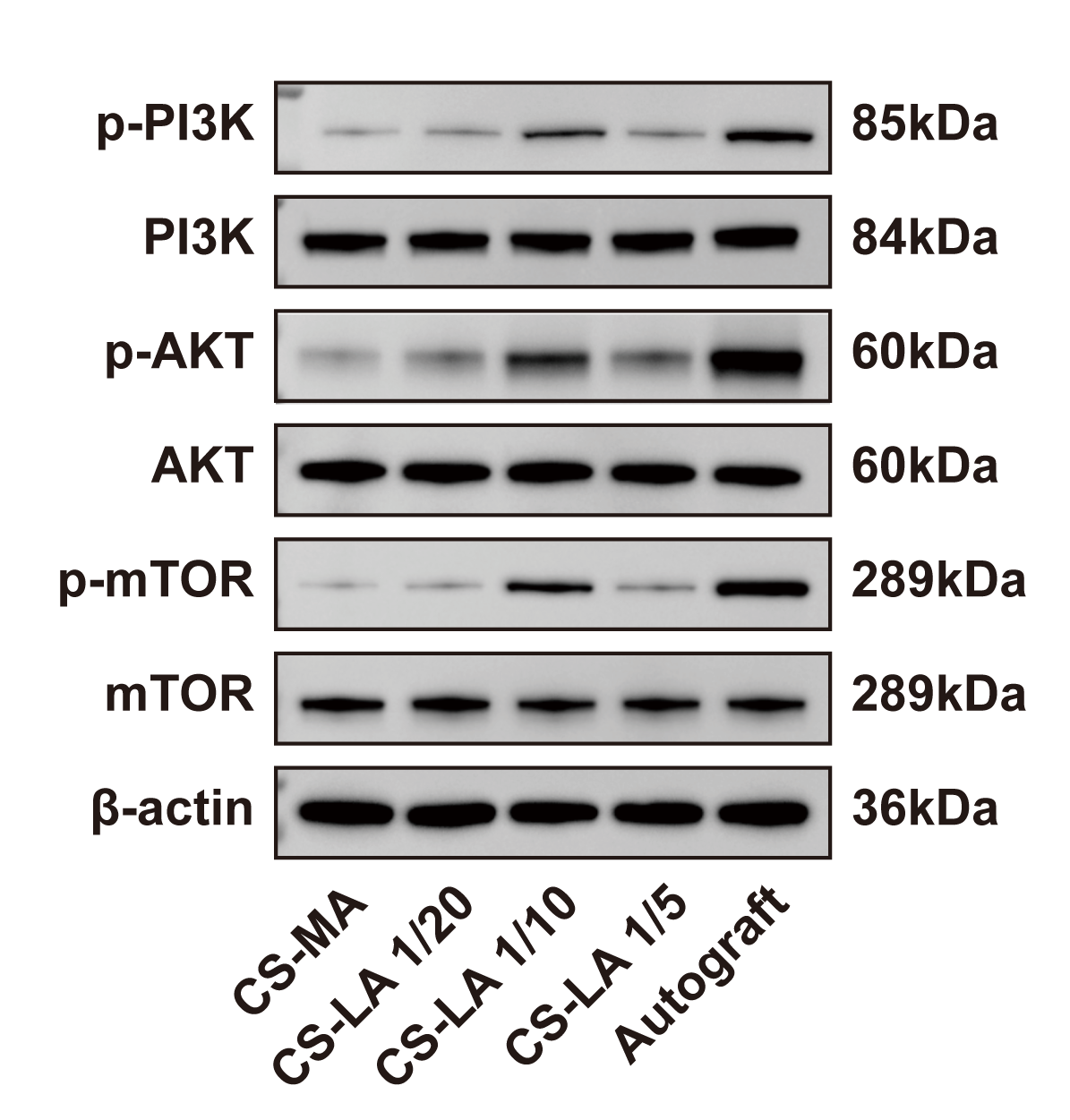


**Figure S9.** Western blot results of protein expression of p-PI3K, PI3K, p-AKT, AKT, p-mTOR and mTOR in proximal nerve stumps at 1st week post-surgery.


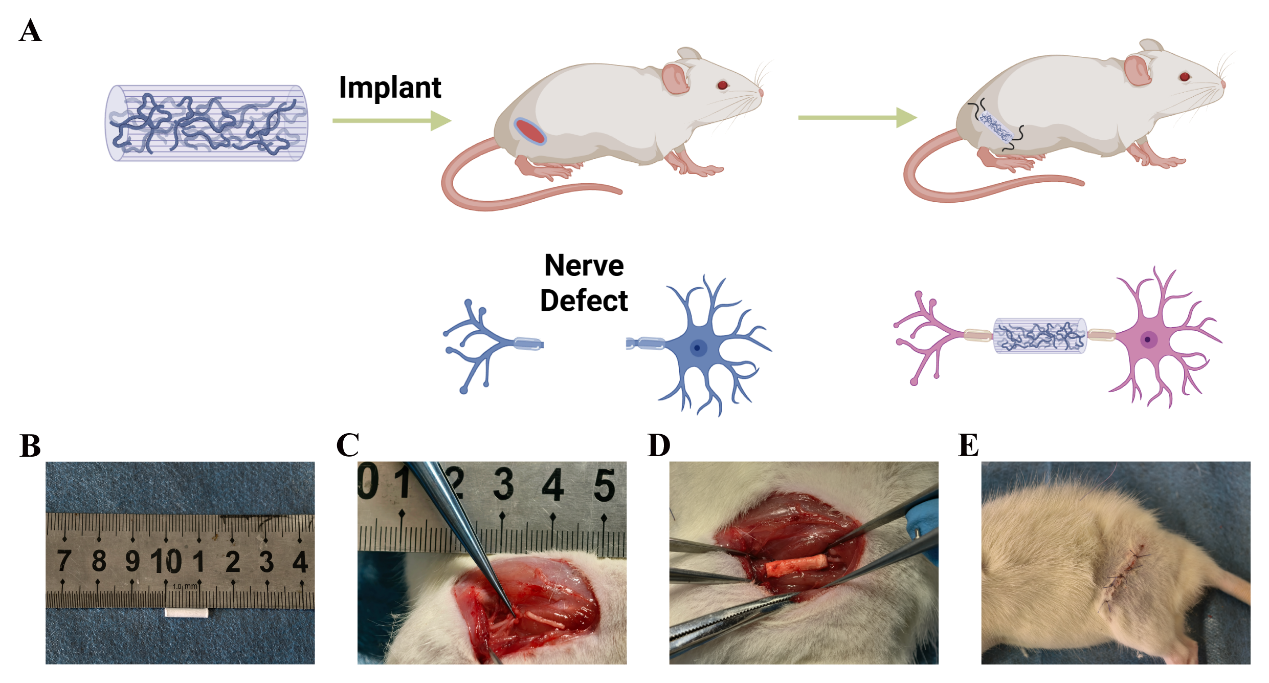


**Figure S10.** (A) The surgical procedure diagram of animal experiment. (B) Appearance and length measurement of CS-LA/P(MMD-CL) conduit. (C–E) Images of the surgical procedure for implanting the CS-LA/P(MMD-CL) conduit into SD rat.


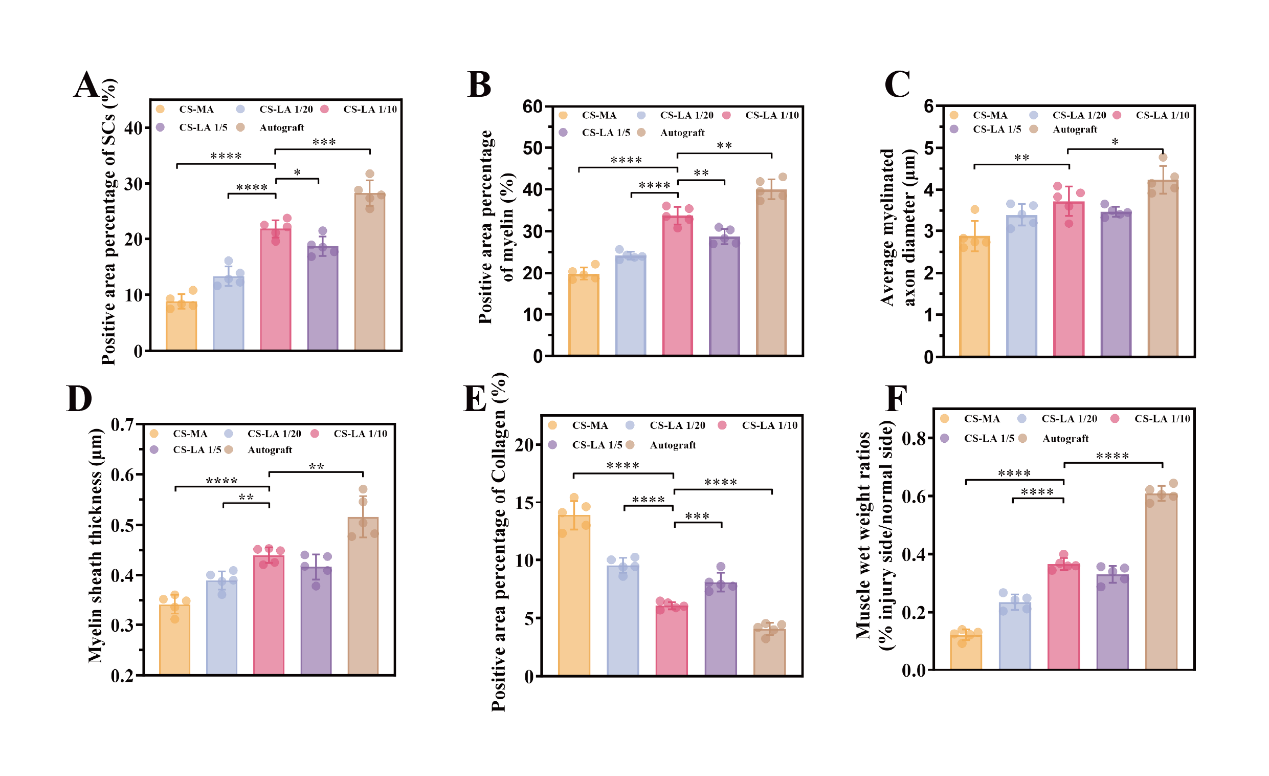


**Figure S11.** (A) The content of Schwann cells in regenerated nerves of different groups (n = 5). (B) Myelin density in regenerated nerves of different groups (n = 5). (C) Statistical data of axon diameter (n = 5). (D) Statistical data of myelin thickness (n = 5). (E) Statistical data of collagen deposition (n = 5). (F) Statistical data of muscle weight loss (n = 5). **P* < 0.05, ***P* < 0.01, ****P* < 0.001, *****P* < 0.0001.


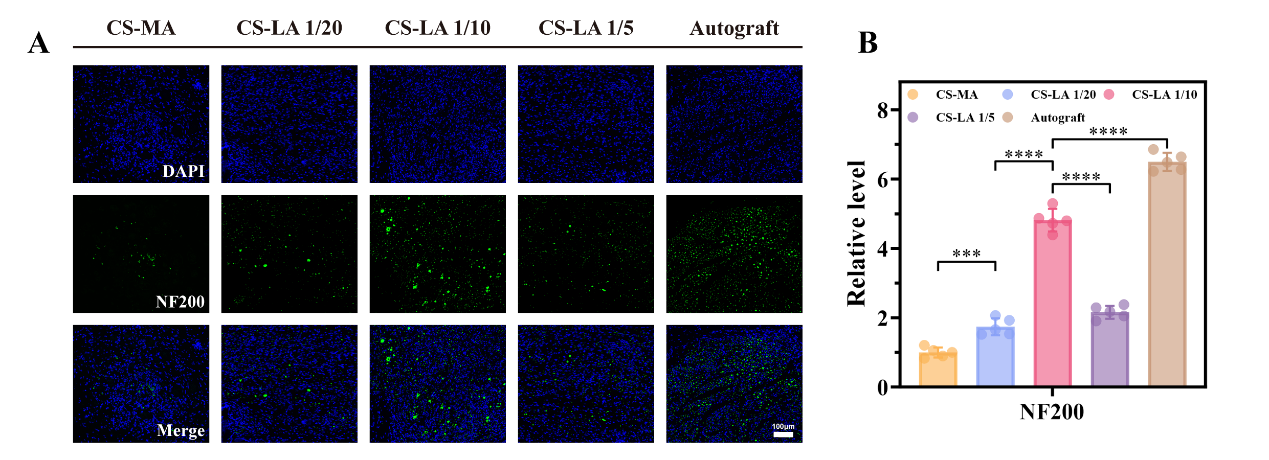


**Figure S12.** (A) Immunofluorescence staining of NF200 on cross-sections of the proximal nerve at 12 weeks post-surgery. Scale bar: 100 μm. (B) Statistical data of NF200 fluorescence staining (n = 5). **P* < 0.05, ***P* < 0.01, ****P* < 0.001, *****P* < 0.0001.


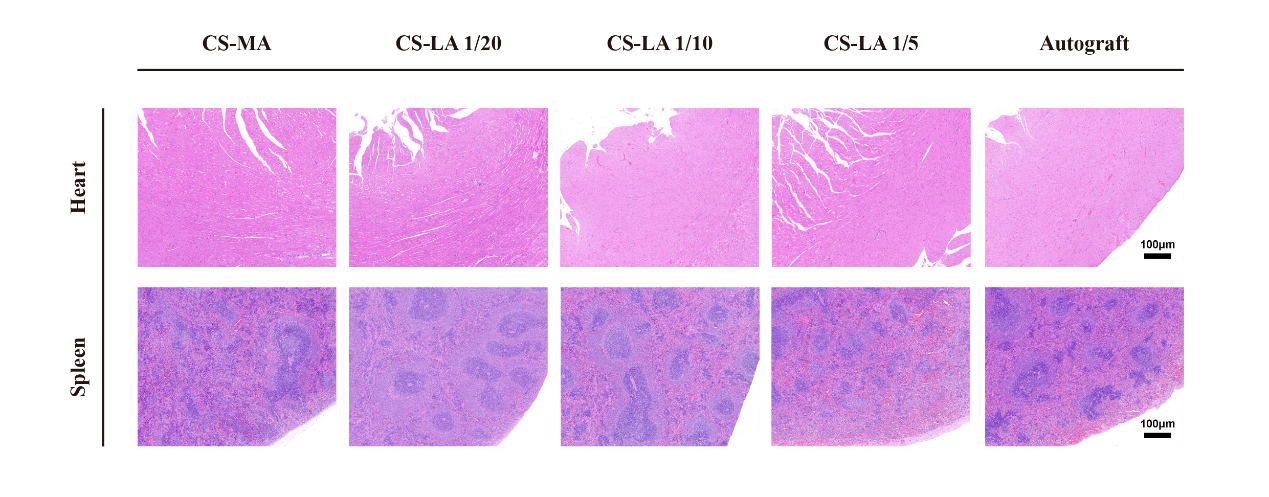


**Figure S13.** H&E staining of the heart and spleen at the 12th week post-surgery. Scale bar: 100 μm.

**Table S1.** Primer sequences for RT-qPCR

| Primer | Sequence | |
| --- | --- | --- |
| Actin | Forward | TCCTTCCTGGGCATGGAGT |
|  | Reverse | AGCACTGTGTTGGCGTACAG |
| CD31 | Forward | AAGTGGAGTCCAGCCGCATATC |
|  | Reverse | ATGGAGCAGGACAGGTTCAGTC |
| VEGF | Forward | TGCAGATTATGCGGATCAAACC |
|  | Reverse | TGCATTCACATTTGTTGTGCTGTAG |
| TNF-α | Forward | GGTGCCTATGTCTCAGCCTCTT |
|  | Reverse | GCCATAGAACTGATGAGAGGGAG |
| IL-10 | Forward | CAGAGCCACATGCTCCTAGA |
|  | Reverse | TGTCCAGCTGGTCCTTTGTT |

**Table S2.** Primer sequences for RT-qPCR

| Primer | Sequence | |
| --- | --- | --- |
| GAPDH | Forward | TGAAGGGTGGAGCCAAAAG |
|  | Reverse | AGTCTTCTGGGTGGCAGTGAT |
| PI3K | Forward | CACGGCGATTACACTCTTACACT |
|  | Reverse | ATCCTGCTGGTATTTGGACACT |
| AKT | Forward | GCTTCTATGGTGCGGAGATTG |
|  | Reverse | CACAGCCCGAAGTCCGTTA |
| Ki67 | Forward | CCTTGCTGAGAACACCACAGC |
|  | Reverse | TTGGGTGACCATCTGACTTCCT |
| mTOR | Forward | AAGCCAAGCCTTGGATTTTG |
|  | Reverse | GGACGGGTGAGGTAACAGGAT |
| IL-10 | Forward | GGTTGCCAAGCCTTATCGG |
|  | Reverse | ACCTGCTCCACTGCCTTGC |
| TNF-α | Forward | CTACTCCCAGGTTCTCTTCAAGG |
|  | Reverse | CTCCCAGGTATATGGGCTCATAC |

**Table S3.** Grafting ratio of lipoic acid on chitosan

|  | **N (%)** | **C (%)** | **H (%)** | **S (%)** | **Grafting ratio of lipoic acid** |
| --- | --- | --- | --- | --- | --- |
| **CS** | 7.06 | 39.03 | 6.79 | 0.00 | 0 |
| **CS-LA 1/20** | 5.80 | 35.01 | 6.78 | 0.42 | 1.37% |
| **CS-LA 1/10** | 5.62 | 35.08 | 6.50 | 1.68 | 5.80% |
| **CS-LA 1/5** | 5.53 | 35.93 | 6.78 | 2.78 | 9.85% |
