# Supplementary material for: ROS-scavenging lipoic acid-modified chitosan hydrogels with rapid photocrosslinked ability accelerates peripheral nerve regeneration
Source: Regen Biomater. 2026 Apr 3;13:rbag068. doi: 10.1093/rb/rbag068 (PMC13110000; doi:10.1093/rb/rbag068)
Supplement: rbag068_Supplementary_Data [file rbag068_supplementary_data.zip › Response_letter.docx]

Dear Editors:

Thank you for your patience in evaluating our manuscript submitted to Regenerative Biomaterials (**Manuscript ID: RB-2025-753**. **TITLE: ROS-scavenging lipoic acid modified chitosan hydrogels with rapid photocrosslinked ability accelerates peripheral nerve regeneration**) and your kindness to provide us the opportunity to revise our manuscript and improve its quality. We also thank all the referees for these constructive comments and suggestions.

Point-by-point responses to the reviewers’ comments are provided on the following pages. According to the reviewers’ constructive suggestions, we have carefully revised the manuscript and made corresponding modifications accordingly. All changes made in the revised version are highlighted in red. We sincerely appreciate your time and effort in handling our manuscript. Below are our detailed responses to each comment. We hope the revised manuscript has been significantly improved, and we are willing to address any additional questions or concerns you may have. The response letter and revised manuscript have been uploaded as separate files.

Yours Sincerely,

Honglian Dai

Ph.D, Doctor

State Key Laboratory of Advanced Technology for Materials Synthesis and Processing, and Hubei Key Discipline Laboratory of Orthopedic Tissue Injury and Repair, Wuhan University of Technology, Wuhan 430070, China

E-mail: daihonglian@whut.edu.cn

Date: March 6, 2026

**Reviewer: 1**

**Comments to the Author**
*The manuscript entitled “ROS-scavenging lipoic acid modified chitosan hydrogels with rapid photocrosslinked ability accelerates peripheral nerve regeneration” tried to addresses the key pathological issues of oxidative stress and excessive inflammatory microenvironment following peripheral nerve injury by designing and constructing an α-lipoic acid-modified chitosan composite hydrogel conduit (CS-LA/P(MMD-CL)) that combines rapid photopolymerization with reactive oxygen species scavenging capabilities. The research topic is well-defined and holds significant clinical relevance and application potential. However, the manuscript falls short in several areas, including the depth of innovation justification, thoroughness of mechanism exploration, and aspects of experimental design and data presentation, leaving it insufficient for direct acceptance before further revison. Specific issues are as follows:*

Response: Thank you very much for your valuable comments to improve our manuscript.

***Comments* 1:** *The validation of CS-LA's pro-angiogenic function in Figure 4 primarily involves endothelial cell migration, and tubule formation assays. This study lacks endothelial cell proliferation data; it is recommended to add this assay to better demonstrate its pro-angiogenic function.*

Response 1: Thank you very much. To further validate the pro-angiogenic function of CS-LA, we have performed an additional CCK-8 assay to assess the proliferation of human umbilical vein endothelial cells. The corresponding results are presented in Supplementary Data (Fig. S6), and the relevant text has been revised accordingly (Page 19). As shown in the updated Fig. S6, the OD values of HUVECs in CS-LA 1/10 were significantly higher than those in control groups at 3 and 5 days; and the OD values of HUVECs in CS-LA 1/20 also significantly higher than those in control groups at 5 days, indicating that CS-LA could promote the proliferation of endothelial cells. This result, combined with our previous data on endothelial cell migration and tubule formation, provides comprehensive evidence that CS-LA effectively enhances the pro-angiogenic function of endothelial cells.

**Figure S6.** The effect of CS-MA and different CS-LA hydrogels on the activity of HUVECs. **P* < 0.05, ***P* < 0.01, ****P* < 0.001, *****P* < 0.0001.

***Comments* 2:** *The representative image from the DCFH-DA staining experiment in Figure 5C only provides a single green fluorescence image without images of labeled cells, which inadequately demonstrates its antioxidant capacity. Please provide the complete images.*

Response 2: Thank you for your valuable suggestion. We have revised the DCFH-DA staining experiment images in Figure 5C (Page 22) to provide a more comprehensive demonstration of the antioxidant capacity of CS-LA. As shown in the updated Figure 5C, we have now included the bright-field images of the cells and merged images alongside the DCFH-DA images. This complete set of images clearly shows the distribution of ROS within the labeled cells, allowing for a more accurate and convincing assessment of the ROS levels in each group.

**Figure 5. Antioxidative effects of CS-LA hydrogel.** (A) The MMP of RSC was detected using JC-1 fluorescent probes. Scale bar: 200 μm. (B) Statistical data of the red and green fluorescence ration in RSC (n = 3). (C) The effect of CS-LA hydrogels on ROS in RSCs. Scale bar: 200 μm. (D) Statistical data of CS-LA hydrogels antioxidant effect (n = 3). **P* < 0.05, ***P* < 0.01, ****P* < 0.001, *****P* < 0.0001

***Comments* 3:** *The background color in the fluorescence image of Figure 5A is quite bright. It is recomended that this image could be adjusted?*

Response 3: Thank you. We have adjusted the fluorescence images in Figure 5A (Page 22) to optimize the background brightness and contrast, ensuring that the experimental results are more clearly and accurately represented. The revised images have been updated in the revised manuscript and are presented above for your reference.

***Comments* 4:** *Figures 7B-E primarily demonstrate that CS-LA/P(MMD-CL) effectively promotes cell proliferation, with the PI3K/AKT/mTOR signaling pathway playing a crucial role in this process. However, relying solely on qPCR data to establish the correlation between cell proliferation and this pathway is insufficient. It is recommended to incorporate Western blot (WB) experiments to better elucidate the involvement of this pathway. Furthermore, this study focuses on ROS scavenging. While it detected upregulation of genes in the PI3K/AKT/mTOR pathway, a direct causal relationship between this pathway and ROS scavenging has not been sufficiently established. Arbitrarily selecting a pathway related to proliferation holds little significance.*

Response 4: We sincerely appreciate your valuable and insightful comments, which have greatly helped us improve the rigor of our manuscript. All revisions and additional data have been incorporated into the revised manuscript and supplementary data, with changes marked in red (Page 24).

1. Regarding the need for Western blot (WB) data to validate the PI3K/AKT/mTOR pathway:

We fully agree that relying solely on RT-qPCR data is insufficient to establish the correlation between cell proliferation and the PI3K/AKT/mTOR pathway. To address this, we have performed additional WB experiments to detect the protein levels of key components and their phosphorylation status in the in vivo nerve regeneration model. As shown in the updated Fig. S9 (Supplementary Data, Page S8), CS-LA/P(MMD-CL) significantly increased the phosphorylation levels of PI3K, AKT, and mTOR, while the total protein levels remained stable. These results are consistent with our RT-qPCR data, providing robust evidence that CS-LA/P(MMD-CL) activates the PI3K/AKT/mTOR pathway at both the gene and protein levels to promote cell proliferation.

2. Regarding the direct causal relationship between ROS scavenging and pathway activation:

To establish a direct link between ROS scavenging and the activation of the PI3K/AKT/mTOR pathway, we designed a series of *in vitro* mechanistic experiments using H_2_O_2_-treated RSC cells (Fig. S8, Supplementary Data, Page S8). First, we confirmed that H_2_O_2_-induced oxidative stress significantly suppressed the phosphorylation of PI3K, AKT, and mTOR. Importantly, treatment with CS-LA effectively reversed this inhibition, restoring the phosphorylation levels of these proteins. Furthermore, the addition of the PI3K/AKT inhibitor LY294002 completely abolished the CS-LA-mediated restoration of p-AKT and p-mTOR levels. These results demonstrate that CS-LA, by scavenging ROS, directly activates the PI3K/AKT/mTOR pathway in a PI3K-dependent manner under oxidative stress conditions. It can be prove that CS-LA/P(MMD-CL) exerts its ROS-scavenging effects, which in turn activate the PI3K/AKT/mTOR signaling cascade to promote cell proliferation.

**Figure S9.** Western blot results of protein expression of p-PI3K, PI3K, p-AKT, AKT, p-mTOR and mTOR in proximal nerve stumps at 1st week post-surgery.

**Figure S8.** Western blot results of protein expression of p-PI3K, PI3K, p-AKT, AKT, p-mTOR and mTOR in RSCs from CON, H2O2, H2O2 + CS-LA 1/10, LY294002, H2O2 + CS-LA 1/10 + LY294002 groups.

***Comments* 5:** *Figure 7F shows that both the CS-MA group and the Autograft group exhibit well-myelinated, striated axonal morphology in the S100 and NF200 fluorescence images. However, quantitative data indicate that the CS-MA group performed poorly. What is the reason for this? Furthermore, these fluorescence images represent neural tissue from the first week post-transplantation. Could you specify the precise location of the tissue depicted? Is it the proximal, distal, or middle segment of the neural tissue?*

Response 5: Thank you very much for your valuable comments and careful observation.

Regarding the discrepancy between the fluorescence images and quantitative data in the CS-MA group: This was indeed an oversight in our initial image processing. The original fluorescence images had low signal intensity, and we only increased the overall brightness without adjusting the contrast. This led to the appearance of non-specific background noise and artifacts, which were incorrectly interpreted as positive staining for S100 and NF200, rather than the actual target proteins. We have now reprocessed all problematic images by optimizing both brightness and contrast to eliminate these artifacts (Fig. 7F, Page 25). We have also re-performed the quantitative analysis based on the corrected images (Fig. 7G, Page 25), which confirmed that our original quantitative conclusions were accurate. During our self-check, we identified a similar image processing issue in Fig. 6F, which has also been corrected and re-analyzed (Fig. 6G, Page 23) to ensure the consistency and reliability of our results.

For the location of the neural tissue in the fluorescence images: All tissue sections used for immunofluorescence staining, including those at week 1 and week 12 post-surgery, were taken from the proximal stumps of the regenerated nerve tissues. This information has been clearly specified in the revised manuscript to ensure experimental transparency.

All revised images have been updated in the manuscript and all revision have been marked in red in the revised manuscript.

**Figure 7. The proliferative effects of CS-LA hydrogel.** (A) Schematic diagram of CS-LA/P(MMD-CL) promotes regenerated axon growth. (B) Immunofluorescence staining of Ki67 on the proximal nerve stumps at 1st week post-surgery. Scale bar: 100 μm. (C) Statistical data of Ki67 fluorescence intensity (n = 5). (D) PI3K and AKT expression at the 1st week post-surgery were obtained by RT-qPCR (n = 5). (E) Ki67 and mTOR expression at the 1st week post-surgery were obtained by RT-qPCR (n = 5). (F) Immunofluorescence staining of NF200/S100 on the proximal nerve stumps at 1st week post-surgery. Scale bar: 100 μm. (G) Statistical data of NF200 and S100 fluorescence staining (n = 5). **P* < 0.05, ***P* < 0.01, ****P* < 0.001, *****P* < 0.0001

**Figure 6. Anti-inflammatory and antioxidative effects of CS-LA hydrogel.** (A) CS-LA hydrogels reduce inflammatory responses in vitro. Scale bar: 400 μm. (B and C) Statistical data of IL-1β and NF-κB fluorescence intensity (n = 3). (D and E) TNF-α and IL-10 expression in vitro cell experiments obtained by RT-qPCR (n = 3). (F) IL-1β/NF-κB Immunofluorescence staining on proximal nerve stumps at 1st week post-surgery. Scale bar: 100 μm. (G) Statistical data of IL-1β and NF-κB fluorescence staining (n = 5). (H) Schematic diagram of CS-LA hydrogel network reacts with ROS reducing oxidative stress of injury microenvironment. **P* < 0.05, ***P* < 0.01, ****P* < 0.001, *****P* < 0.0001

***Comments* 6:** *Figure 8J Autograft group: Why is the cell count particularly low in the first DAPI image? (Uncertain if this is an image clarity issue) CS-LA 1/20 NF200 image: The background is also very bright? Does it need adjustment?*

Response 6: We sincerely appreciate your careful observation and valuable comments on Figure 8J. The low cell count in the Autograft DAPI image was indeed due to image clarity issues. We have reprocessed this image using ImageJ (Ver. 1.8.0) to optimize brightness and contrast to improve the quality of images. The high-resolution original image file has also been uploaded to assist you in further evaluating the image quality. Similarly, we have adjusted the background brightness of the CS-LA 1/20 NF200 image to reduce the background noise and improve the overall clarity. All revised images have been updated in the manuscript (Page 27).

**Figure 8. The axonal morphology and nerve function recovery effect of CS-LA/P(MMD-CL).** (A) Stained images of HE, LFB and TB. Scale bars: 50 μm, 100 μm, 50 μm. (B) Images of the structure of mature peripheral nerve. (C) TEM images of transverse sections of regenerated nerves. Scale bars: 2 μm, 1 μm, 200 nm. (D) Typical image of the rat footprints collection device. (E) Schematic diagram of rat footprints (normal side: black; injured side: red). (F) SFI values of rats in the 12th week (n = 5). (G) Muscle contrast images (left: injured side; right: normal side) and Masson staining of the injured side at 12th week post-surgery. Scale bar: 50 μm. (H) H&E staining of the liver and kidney at 12th week post-surgery. Scale bar: 100 μm. (I) Schematic diagram of metabolic pathways of degradation by-products of CS-LA/P(MMD-CL). (J) Immunofluorescence staining of NF200/S100 on longitudinal sections of the proximal nerve stumps at 12th week post-surgery. Scale bar: 100 μm. (K) Statistical data of NF200 and S100 fluorescence staining (n = 5). **P* < 0.05, ***P* < 0.01, ****P* < 0.001, *****P* < 0.0001

***Comments 7:*** *In the description of the neural tissue section from week 12, the authors did not specify the location of the section—was it from the proximal, distal, or middle end of the neural tissue? The manuscript also includes fluorescence images from week 1 of the animal experiments. Where exactly was the neural tissue section taken in week 1? Was it from the proximal, distal, or middle end of the neural tissue?*

Response 7: We sincerely appreciate your valuable suggestion and apologize for our oversight in not clearly specifying the location of neural tissue sections in the manuscript. Our study aims to investigate the regeneration of peripheral nerve tissue after ROS scavenging by CS-LA. Therefore, all pathological tissue stainings and fluorescence images (including those at week 1 and week 12 post-surgery) were obtained from sections of the proximal end of the neural tissue. We have now supplemented this information in all relevant sections of the revised manuscript and all changes are marked in red.

***Comments* 8:** *In the animal surgery section, the authors' description is overly simplistic. Information provided about the prepared nerve conduit only mentions a diameter of 1.5 mm, without specifying its length—a critical factor for constructing specific nerve injury models in animal experiments. The authors merely state that this is a nerve transection model without further elaboration, which could be misinterpreted as a nerve compression model, potentially affecting subsequent results. It is recommended that the authors include images of the surgical procedure to better illustrate the situation.*

Response 8: We appreciate your valuable suggestion. We have supplemented the animal surgery section with more detailed information, which is now included in Supplementary Data (Fig. S10, Supplementary Data) (Page S4-5 & S9).

SD rats were anesthetized with 10% chloral hydrate (400 mg/kg, i.p.) and fixed in a prone position. A longitudinal incision was made on the hind limb, and the sciatic nerve was exposed. A 10 mm segment of the sciatic nerve was excised to create a standardized 10 mm sciatic nerve transection defect model, clearly distinguishing it from a nerve compression model. The prepared nerve conduits or autologous nerve grafts were sutured to the nerve stumps with 8-0 PLGA sutures, followed by closure of the muscle and skin with 4-0 PLGA sutures. Postoperatively, the incision was disinfected, and the rats were housed with enough water and food.

**Figure S10.** (A) The surgical procedure diagram of animal experiment. (B) Appearance and length measurement of CS-LA/P(MMD-CL) conduit. (C–E) Images of the surgical procedure for implanting the CS-LA/P(MMD-CL) conduit into SD rat.

***Comments* 9:** *This study only characterized longitudinal sections of neural tissue. We recommend that the authors supplement their findings with representative cross-sectional images of NF200 staining in neural tissue, as this would more effectively demonstrate neural regeneration.*

Response 9: Thank you for your suggestion. We have added the cross-sectional images of NF200 staining of the proximal nerve (Fig. S12, Supplementary Data, Page S10) in the revised manuscript (Page 26). The Quantitative analysis of the results showed that CS-LA/P(MMD-CL) can improve the repair level of regenerated nerves and the highest improvement observed was CS-LA 1/10, which is consistent with the finding of longitudinal sections of the nerve tissues.

**Figure S13.** (A) Immunofluorescence staining of NF200 on cross-sections of the proximal nerve at 12 weeks post-surgery. Scale bar: 100 μm. (B) Statistical data of NF200 fluorescence staining (n = 5). **P* < 0.05, ***P* < 0.01, ****P* < 0.001, *****P* < 0.0001.

**Reviewer: 2**

**Comments to the Author**
*This study developed a CS-LA/P(MMD-CL) composite nerve guidance conduit for peripheral nerve injury. The system effectively alleviates oxidative stress, regulates the inflammatory microenvironment, and promotes angiogenesis, thereby facilitating peripheral nerve regeneration and functional recovery. The experimental design covers the entire process from material synthesis, physicochemical characterization, in vitro cell experiments to in vivo rat sciatic nerve defect models. However, the manuscript still has room for improvement, and several issues require the authors to fully respond to:*

Response: Thank you very much for your significant comments to help us improve the quality of this manuscript.

***Comments* 1:** *The abstract emphasizes the challenges faced by anti-inflammatory drug-delivering hydrogels, but the advantages of the CS-LA hydrogel in this regard are not adequately described. Additionally, the specific advantages of the composite system of CS-LA and aligned P(MMD-CL) conduit are not clearly articulated. It is recommended to streamline some redundant mechanistic descriptions, highlight the core innovations, and enhance the conciseness of the abstract.*

Response 1: Thank you. In line with your suggestions, we have thoroughly revised the abstract. We clearly distinguished our CS-LA hydrogel from traditional drug-loaded hydrogels that rely on sustained anti-inflammatory drug release. While the CS-LA hydrogel achieves intrinsic ROS-scavenging and anti-inflammatory effects via the high reactivity of its disulfide bonds, eliminating the need for loading exogenous anti-inflammatory drugs and the subsequent requirement for precisely engineered hydrogel systems to match drug release kinetics. Moreover, it enables photoinitiator-free rapid crosslinking under 365 nm UV irradiation, avoiding potential cytotoxicity from additional photoinitiators. Then, we clarified the synergistic structural and functional advantages of the composite system. For the CS-LA hydrogel, it modulates the pathological PNI microenvironment by scavenging excessive ROS, alleviating oxidative stress and resolving excessive inflammation; for the oriented P(MMD-CL) conduit, it provides robust physical support and directional guidance for regenerating axons. This simple yet integrated composite design avoids the complexity of traditional multi-component conduit systems and the associated risks of cytotoxicity, realizing a synergistic effect of microenvironment modulation and structural guidance for efficient peripheral nerve regeneration. Finally, We have deleted redundant mechanistic descriptions irrelevant to the core design of the composite system, and centered the abstract on its core innovations. We believe that the revised version well addresses the concerns you raised with improved clarity and conciseness.

***Comments* 2:** *The rationale for the dimensions of the synthesized P(MMD-CL) conduit (1.5 mm in diameter and 14 mm in length) in the "Materials and Methods" section is not clearly explained. It is suggested to supplement descriptions regarding the compatibility of these dimensions with the SD rat sciatic nerve defect model to enhance the rationality of the experimental design.*

Response 2: Thank you for your suggestion. The dimensions of the P(MMD-CL) conduit were strictly designed for the sciatic nerve injury model of adult SD rats used in this study, matching the anatomical characteristics and injury model design of the rat sciatic nerve. The inner diameter of 1.5 mm of the conduit is consistent with the average transverse diameter of the sciatic nerve in adult SD rats (approximately 1.5 mm), ensuring close fitting of the conduit with the nerve stumps and avoiding excessive space that may lead to fibrous tissue hyperplasia. Meanwhile, we corrected the writing error in the original text. The actual length of all CS-LA/P(MMD-CL) conduits is approximately 12 mm. The 1 mm redundant length at both ends of the conduit is specially designed to facilitate the surgical suture and fixation between the conduit and the nerve stumps, ensuring the stability of the conduit implantation and the continuity of regenerated axon growth. All the above supplementary explanations and writing corrections have been completed in the revised supplementary data, with the relevant content marked in red (Page S2).

***Comments* 3:** *In the mechanism schematic (Figure 1), the association between certain signaling pathways (e.g., PI3K, NF-κB) and the role of CS-LA is not clearly labeled. It is recommended to optimize the arrow directions and labeling logic of the diagram to make the relationships among oxidative stress, inflammatory regulation, and nerve regeneration signaling pathways more intuitive and understandable.*

Response 3: Thank you. We have optimized the arrow directions and labeling logic to explicitly show the mechanistic cascade and have updated the corresponding paragraph in the manuscript to align with the optimized schematic (Page 5). We believe These revisions make the mechanistic relationships among oxidative stress, inflammatory regulation, and nerve regeneration more transparent and understandable, and could improve the clarity of our work.

**Figure 1. Schematic diagram of the mechanism of CS-LA/P(MMD-CL) conduit construction.** After PNI, apoptosis and immune system trigger excessive ROS release, creating a microenvironment of oxidative stress and excessive inflammation. The S-S of CS-LA hydrogel react with and scavenge ROS, thereby downregulates pro-inflammatory factors expression and upregulates restorative factors (e.g., IL-10, VEGF) secretion, which activates the PI3K/AKT pathway to accelerate cell proliferation and angiogenesis, provide nutrient support, and ultimately promote axonal regeneration and motor function recovery.

***Comments* 4:** *In Results and Discussion section, only the role of the blood supply system in peripheral nerve regeneration and the impact of the material on blood supply reconstruction are elaborated. The specific mechanism by which inflammatory microenvironment regulation influences angiogenesis is not briefly explained. It is suggested to supplement relevant interpretations of the mechanistic pathways through which inflammatory microenvironment regulation promotes angiogenesis.*

Response 4: Thank you. We fully agree with your suggestion and have supplemented the relevant mechanistic interpretation in the Cytotoxicity and angiogenesis studies section. As elaborated in the revised manuscript, the CS-LA component in the composite conduit exerts efficient ROS-scavenging activity, which modulates the inflammatory microenvironment and would promote M1-to-M2 macrophage polarization theoretically (*Nature Reviews Immunology 2009;9:259-270; Acta Neuropathologica 2015;130:605-618*); the polarized M2 macrophages secrete VEGF-A, and this pro-angiogenic factor is synergistically supplemented by VEGF-A released from Schwann cells. The combined VEGF-A activates the PI3K-Akt signaling pathway in vascular endothelial cells, and this process is further reinforced by the upregulated CD31 expression, collectively mediating pro-angiogenic effect (Page 20).

***Comments* 5:** *In Results and Discussion section, the CS-LA 1/10 group exhibits the optimal nerve repair effect, but the specific mechanism underlying the "inhibited repair efficiency in the group with excessively high LA ratio (1/5 group)" is not briefly explained. It is recommended to supplement speculations or relevant explanations regarding how excessively high LA concentrations may affect the homeostasis of the cellular microenvironment to make the result analysis more comprehensive.*

Response 5: Thank you, we appreciate your suggestion to clarify this mechanism. As a naturally occurring mitochondrial cofactor for key metabolic enzymes (e.g., pyruvate dehydrogenase), α-lipoic acid (ALA) is safe at appropriate doses but exhibits concentration-dependent effects. Based on existing reports of adverse reactions from excessive ALA intake and our experimental observations, we speculate that the excessively high LA concentration in the 1/5 group disrupts intracellular redox homeostasis and inhibits mitochondrial enzyme activity, leading to reduced ATP synthesis. This metabolic imbalance impairs the proliferation and paracrine function of functional cells (e.g., Schwann cells and vascular endothelial cells) involved in nerve repair, ultimately offsetting ALA’s antioxidant benefits and resulting in inferior repair outcomes. We plan to verify this proposed mechanism through targeted experiments like assessing mitochondrial function in future studies to provide more direct evidence.

**Reviewer: 3**

**Comments to the Author**
*This study focuses on peripheral nerve injury repair and constructs a CS-LA/P(MMD-CL) composite nerve guidance conduit loaded with an antioxidant chitosan hydrogel. The repair efficacy of the material has been systematically verified through material synthesis, physicochemical characterization, in vitro cell experiments, and an in vivo SD-rat sciatic nerve defect model. The experimental design is comprehensive, the data are detailed and reliable, and the research logic is clear. The core conclusions of the manuscript are clear, and the existing experimental data are sufficient to support the statements. However, the authors still need to address minor issues in expression and logical organization to enhance the academic rigor and readability of the manuscript.
Here are some specific comments:*
Response: We greatly appreciate the constructive comments and valuable suggestions, which have significantly help us improve the quality of this manuscript.

***Comments* 1:** *The abstract insufficiently discusses the core characteristics of the composite conduit, with some problems of disordered phrasing. In addition, the presentation of crucial in vivo experimental results (such as key indicators, including nerve function recovery scores and axon regeneration density) is not sufficiently concise and clear. It is recommended to reorganize the abstract's logic flow, elaborate on the structural advantages and core functions of the composite conduit, concisely present the key experimental conclusions, remove redundant descriptions irrelevant to the core innovations, and improve the logicality and information density of the abstract.*
Response 1: Thank you very much. In accordance with your suggestions, we have thoroughly revised the abstract. In the revised abstract, we have clearly delineated the critical limitations of conventional drug-loaded nerve conduits (complex hydrogel engineering for matching drug release kinetics, potential cytotoxicity from complex systems, and aseptic inflammation induced by acidic degradation products), then explicitly elaborated the core structural and functional features of our composite conduit, which integrated a photoinitiator-free CS-LA hydrogel with rapid 365 nm UV photo-crosslinking and intrinsic ROS-scavenging capacity into an oriented P(MMD-CL) conduit that provides physical support and directional guidance for axon growth. The synergistic functional mechanism of the two components was also clearly stated to rationalize the narrative and highlight the conduit’s core attributes. Moreover, We have concisely incorporated the critical quantitative indicator of nerve function recovery (sciatic functional index, SFI: -77.51 ± 1.51) as a core in vivo outcome, and summarized other key experimental conclusions, including enhanced cell proliferation and angiogenesis, improved maturity of regenerated peripheral nerves, mitigation of gastrocnemius atrophy, and great biocompatibility. Finally, we have deleted all redundant descriptions irrelevant to the core innovation of the composite conduit. The revised abstract now focuses more on the core design, structural advantages, key functional effects and critical experimental outcomes of the CS-LA/P(MMD-CL) composite conduit and we believe the revised version fully addresses the concerns you raised.

***Comments* 2:** *Writing issues: After defining abbreviations in the text (e.g., PNI, UV, S-S), the full names should not be used again; that is to say, the abbreviations should be used throughout the manuscript to improve its readability.*
Response 2: Thank you very much for your valuable comment on the manuscript’s writing norms. In accordance with your suggestion, we have thoroughly revised the entire manuscript. All terms with defined abbreviations (including PNI, UV, S-S, and other relevant terms) are uniformly expressed by their abbreviations throughout the text after the first full-name definition, and the redundant use of full names has been eliminated. All relevant modifications have been marked in red in the revised version.

***Comments* 3:** *The manuscript fails to specify the statistical methods and test methods used for data analysis, as well as how multiple comparison corrections (e.g., Bonferroni correction or Tukey's test) were performed. This is particularly important considering the large number of indicators evaluated.*
Response 3: Thank you. We have added a dedicated “Statistical Analysis” subsection in the Materials and Methods section to clarify all analytical details (Page 13). This revised text explicitly specifies the software used, the pre-testing of data assumptions, the primary statistical test (one-way ANOVA), the post hoc test (Tukey’s HSD) for multiple comparisons, and the criteria for statistical significance. These revisions ensure that our data analysis methodology is fully transparent and reproducible. All revisions are marked in red in the revised manuscript.

***Comments* 4:** *The in vitro antioxidant experiment only uses the DPPH free radical scavenging method, and relying on a single method limits its persuasiveness. It is recommended to add verification of specific effects on different reactive oxygen species (ROS) (e.g., ·OH, O2-) to support the ROS scavenging function of CS-LA.*
Response 4: Thank you for your valuable suggestion regarding the antioxidant activity assessment. We fully agree that a single DPPH assay is insufficient to fully demonstrate the ROS-scavenging capacity of CS-LA. To comprehensively validate the antioxidant mechanism, we have additionally performed a hydroxyl radical (·OH) scavenging assay to complement the DPPH results. As shown in the updated Supplementary Data (Fig. S4, Page S6), within 6 hours, CS-LA 1/5 exhibited a high ·OH scavenging efficiency of 91.42%, while CS-LA 1/10 and CS-LA 1/20 achieved efficiencies of 79.43% and 61.85%, respectively. These values were significantly higher than the 30.61% efficiency of the CS-MA group. These new data, combined with our previous DPPH results, provide robust evidence that CS-LA hydrogels possess potent and broad-spectrum ROS-scavenging capabilities against both DPPH and ·OH radicals (Page 16-17).

**Figure S4.** Scavenging efficiency of ·OH of CS-LA and CS-MA hydrogels (n = 3).

***Comments* 5:** *Figure referencing corrections: Fig. 1D on page 16 should be Fig. 2D; Fig. 6E on page 26 should be Fig. 7E; Figs. 8B, 8H, and 8I are not referred to in the text.*
Response 5: We apologize for the errors in figure referencing. We have carefully checked and corrected all figure labels in the manuscript and descriptions corresponding to Figs. 8B (Line 622), 8H, and 8I (Line 671) have been added into the main text. All corrections have been marked in red in the revised manuscript.

***Comments* 6:** *In the conclusion section, the potential impact of the research results on biomedicine can be discussed more thoroughly, emphasizing the advantages and limitations of the CS-LA hydrogel compared to other hydrogel materials.*

Response 6: Thank you for your valuable suggestion. We fully agree with your comment and have revised the conclusion section accordingly to address this point comprehensively. In the revised conclusion, we emphasized that oxidative stress and excessive inflammation are common pathological hallmarks across multiple diseases, which extends the application potential of the CS-LA hydrogel beyond PNI repair. We specifically cited potential applications in diabetic wound healing, hypoxic/infected wounds, and other related disease scenarios, highlighting the broad biomedical value of our ROS-responsive and anti-inflammatory design. And we explicitly outlined the key advantage of the CS-LA hydrogel: its ability to undergo rapid photocrosslinking under 365 nm UV light, enabling in-situ gelation that conforms to the irregular shape of injury sites, which is beneficial for clinical application. We believe that this revision enhances the depth of discussion on the research’s biomedical implications and clearly differentiates the CS-LA hydrogel from other hydrogel materials, making the conclusion more comprehensive and insightful (Page 29).
